# Discovering useful genetic variation in the seed parent gene pool for sorghum improvement

**DOI:** 10.3389/fgene.2023.1221148

**Published:** 2023-09-18

**Authors:** Neeraj Kumar, J. Lucas Boatwright, Sirjan Sapkota, Zachary W. Brenton, Carolina Ballén-Taborda, Matthew T. Myers, William A. Cox, Kathleen E. Jordan, Stephen Kresovich, Richard E. Boyles

**Affiliations:** ^1^ Advanced Plant Technology, Clemson University, Clemson, SC, United States; ^2^ Department of Plant and Environmental Sciences, Clemson University, Clemson, SC, United States; ^3^ Carolina Seed Systems, Darlington, SC, United States; ^4^ Pee Dee Research and Education Center, Clemson University, Florence, SC, United States; ^5^ Feed the Future Innovation Lab for Crop Improvement, Cornell University, Ithaca, NY, United States

**Keywords:** DArT markers, genome-wide association studies (GWAS), multi-parent advanced generation inter-cross (MAGIC), grain sorghum, yield components

## Abstract

Multi-parent populations contain valuable genetic material for dissecting complex, quantitative traits and provide a unique opportunity to capture multi-allelic variation compared to the biparental populations. A multi-parent advanced generation inter-cross (MAGIC) B-line (MBL) population composed of 708 F_6_ recombinant inbred lines (RILs), was recently developed from four diverse founders. These selected founders strategically represented the four most prevalent botanical races (kafir, guinea, durra, and caudatum) to capture a significant source of genetic variation to study the quantitative traits in grain sorghum [*Sorghum bicolor* (L.) Moench]. MBL was phenotyped at two field locations for seven yield-influencing traits: panicle type (PT), days to anthesis (DTA), plant height (PH), grain yield (GY), 1000-grain weight (TGW), tiller number per meter (TN) and yield per panicle (YPP). High phenotypic variation was observed for all the quantitative traits, with broad-sense heritabilities ranging from 0.34 (TN) to 0.84 (PH). The entire population was genotyped using Diversity Arrays Technology (DArTseq), and 8,800 single nucleotide polymorphisms (SNPs) were generated. A set of polymorphic, quality-filtered markers (3,751 SNPs) and phenotypic data were used for genome-wide association studies (GWAS). We identified 52 marker-trait associations (MTAs) for the seven traits using BLUPs generated from replicated plots in two locations. We also identified desirable allelic combinations based on the plant height loci (*Dw1*, *Dw2*, and *Dw3*), which influences yield related traits. Additionally, two novel MTAs were identified each on Chr1 and Chr7 for yield traits independent of dwarfing genes. We further performed a multi-variate adaptive shrinkage analysis and 15 MTAs with pleiotropic effect were identified. The five best performing MBL progenies were selected carrying desirable allelic combinations. Since the MBL population was designed to capture significant diversity for maintainer line (B-line) accessions, these progenies can serve as valuable resources to develop superior sorghum hybrids after validation of their general combining abilities via crossing with elite pollinators. Further, newly identified desirable allelic combinations can be used to enrich the maintainer germplasm lines through marker-assisted backcross breeding.

## Introduction

Sorghum was domesticated in Africa circa 3000 B.C.E., most likely in the Sahel area, where it is one of the most important cereal crops due to its drought tolerance (Kebede, 1991; Ayana and Bekele, 1998). Secondary centers of domestication include India, Sudan, and Nigeria (Ayana and Bekele, 1998). Cultivated sorghum is commonly classified into five main botanical races: bicolor, caudatum, durra, guinea, and kafir (Harlan and de Wet, 1972; Barnaud et al., 2008). These classifications are mostly based on panicle morphology and grain characteristics with additional consideration for the regions of Africa and India where the races are predominantly found (Murray et al., 2009). As with other crops, grain yield in sorghum is a complex trait that is mediated by many genes (Holland, 2007; Boyles et al., 2017b).

Sorghum, like its close relative maize, is primarily grown as a hybrid crop in developed countries. Commercial F_1_ hybrid seed production is dependent on the cytoplasmic male sterility (CMS) system for cross-fertilization. In the CMS system, three distinct line types (A-, B-, and R-lines) are required, and crossing is performed using specific parental pairs (A/B, and R) to produce a hybrid seed (Xin et al., 2021). A_1_ CMS is the predominant sterility source for commercial hybrid seed production in sorghum although other sources do exist in sorghum (A_2–6_ and 9E) (Schertz et al., 1997). The A/B parental group represents the female parent, and crossing proceeds with the A-line (female), which is crossed with a restorer parent (R-line) to produce a hybrid seed (Rooney, 2004). The B-line is a non-restorer, or maintainer, line that perpetuates the male-sterile (A-line) line via backcrossing. This is a time-intensive process that serves to transfer donor cytoplasm and recover the recipient parent genome (Jordan et al., 2010; Mindaye et al., 2015). To date, germplasm development has largely focused on R-lines with publicly available A/B line germplasm being underrepresented in the National Plant Germplasm System (NPGS) (Menz et al., 2004; Xin et al., 2021), which is a function of most sorghum genotypes being partial or full fertility restorers based on one or more nuclear restoration genes.

In the last two decades, the accessibility of relatively inexpensive genotyping costs makes it more effective to study complex traits through association studies (Unterseer et al., 2014; Boatwright et al., 2022). Development of genetic mapping populations represents the most resource-intensive step as a selection of the founder lines and subsequent crossing dictates the number and resolution at which QTL can be identified. The most popular genetic mapping populations are biparental recombinant inbred lines (RILs), F_2_s, doubled haploid (DH), and backcrosses. Among these populations, RILs and DHs have the distinct advantage that they are “immortal” and can be used in multiple experiments. Conversely, RILs and DHs that are derived from biparental crosses exhibit relatively low genetic recombination and diversity with a high probability for parents to carry the same alleles at a locus. To overcome these limitations, multi-parent genetic populations like MAGIC and nested association mapping (NAM) have been established in various crops.

The concept of a MAGIC population has been discussed earlier by Mackay and Powell (2007) and a MAGIC population for the first time was developed in a model crop like *Arabidopsis* (Cavanagh et al., 2008). In the design of the MAGIC population, multiple founders can be intercrossed in a well-defined order in multiple generations to recombine genetic material from founders to develop recombinant lines (Cavanagh et al., 2008). Later, several MAGIC panels were developed in several non-model crops and used for QTL discoveries including wheat (Huang et al., 2012; Mackay et al., 2014), rice (Bandillo et al., 2013), tomato (Pascual et al., 2015), fava bean (Sallam and Martsch, 2015), maize (Dell’Acqua et al., 2015), barley (Sannemann et al., 2015), cowpea (Huynh et al., 2018), sorghum (Ongom and Ejeta, 2018), soybean (Hashemi et al., 2022) and eggplant (Mangino et al., 2022). As a result, MAGIC populations represent an ideal genetic construct to identify favorable alleles from diverse parental lines that can dissect the genetic variation underlying complex, quantitative traits (Mackay and Powell, 2007; Cavanagh et al., 2008; Ongom and Ejeta, 2018). Compared to traditional biparental crosses, MAGIC populations can be used to perform high-resolution genetic mapping because higher recombination rates resulted in faster LD decay (Cavanagh et al., 2008). Multi-parent populations also reduce the effects of confounding due to population structure based on sampling effects (Flint-Garcia et al., 2005; Vilhjálmsson and Nordborg, 2013), which is preferable as population structure can enhance the risk of detecting false positives (Ewens and Spielman, 2001; Dickson et al., 2010; Korte and Farlow, 2013). Using MAGIC populations, a greater number of traits can be targeted depending on the selection of contrasting parental lines involved in the construction of the population.

In the present study, a recently developed MBL population was leveraged to mine the B-line (female) parent gene pool for novel and favorable genetic variation (Kumar et al., 2023). A set of polymorphic, quality-filtered markers (3,751 SNPs) were generated using Diversity Arrays Technology sequencing (DArTseq). GWAS were employed to identify MTAs using genomic data (3,751 SNPs) and phenotypic data for seven traits: PT, DTA, PH, GY, TGW, TN, and YPP. The MBL population consisted of F_6_ RILs derived from an intercross among the four diverse grain sorghum founders. Several significant MTAs associated with the above phenotypic traits were identified, which represent both novel and previously identified genetic loci. In addition, we identified desirable allelic combinations and pleiotropic MTAs (shared associations) between plant height and yield component traits. In this study, we selected best-performing lines based on the new allelic combinations as the unique genetic resources for future breeding efforts to facilitate the pyramiding of desirable alleles using marker-assisted selection. These selected B-lines can be used directly in a hybrid breeding program after validation of general combining abilities.

## Materials and methods

### MBL development and phenotyping

MBL population was developed from crosses between four founder lines SC630 (PI533937), SC605 (PI534096), BTx642 (PI656029), and BTxARG-1 (PI561072). These founders were selected to capture genetic diversity across multiple botanical races (SC630 = kafir, SC605 = guinea, BTx642 = durra, and BTxARG-1 = caudatum) as well as broad phenotypic variation across various qualitative, agronomic and yield related traits (Kumar et al., 2023). In addition, the MBL was genetically characterized for some well-known, heritable traits such as seed color, plant color, and awns using QTL mapping and GWAS. Full details on the development and initial characterization of the MBL panel are described by Kumar et al. (2023).

As previously mentioned, a set of 708 F_6_ RILs and the four founder lines were phenotyped for PT, DTA, PH, GY, TGW, TN, and YPP. The first and last plants in each row were not phenotyped since they served to eliminate confounding results caused by border effects. Panicle type (PT) was visually assessed (C = compact, SC = semi-compact, SO = semi-open, and O = open) at physiological maturity at a single field location (Simpson Research Farm, Pendleton, SC) and used in the analysis as numerical values in 1–4 scales, (1 = compact, 2 = semi-compact, 3 = semi-open, and 4 = open). DTA was measured as days after planting to when 50% of the plants in the plot were at mid-bloom. PH was measured at physiological maturity in centimeters from the ground to the apex of the primary panicle. The tiller number per plot (TN) was estimated by counting the total number of plants from a representative 1-m row. All panicles were harvested from this 1-m section and subsequently threshed to process grain yield (GY) and individual yield component traits. Harvested panicles were dried for 3–4 days in an electric dryer to a constant moisture content (∼12% moisture) and threshed individually with a BT-14E belt thresher (Almaco, Nevada, IA, United States). GY was estimated as a total grain weight of 1-m harvested plot, which was also used for estimating TGW by counting 1,000 grains of every individual using Model U electric seed counters (International Marketing and Design Co., San Antonio, TX, United States). To estimate the grain yield per panicle (YPP), GY was divided by number of panicles. All seven traits were divided into three major categories: 1) panicle morphology (PT), 2) agronomic (DTA and PH) and 3) yield related traits (GY, TGW, TN, and YPP).

### Field trial and maintenance

MBL (708 F_6_) RILs along with their four founders were grown at two field locations including Simpson Research Farm, Pendleton, SC near to Clemson University (designated as CU) and Pee Dee Research and Education Center, Florence, SC (designated as FL) during summer 2021. The first location was planted on 2 June 2021 at Simpson Research Farm (34.624954, −82.726496) in Pendleton, SC included 701 RILs, while the second location was planted on 7 June 2021 at the Pee Dee Research and Education Center (34.287834, −79.744063) in Florence, SC, which included 708 RILs. An alpha lattice field design was used across locations to evaluate the MBL RILs and founder lines, with two replications in each location and four incomplete blocks per replicate. Each incomplete block contained 182 RILs, four founders, and a F_1_ hybrid check. Each genotype was grown as a single-row plot at 3 m in length and a row spacing of 0.76 m. A plant density of ∼130,000 plants ha^−1^ was calculated based on a plant establishment of 75% using a seeding rate (El Naim et al., 2012).

Before planting the field trials, the seeds were treated with a blend of fluxofenim (Concep, herbicide antidote), clothianidin (Nipsit, insecticide), mefenoxam (Apron XL, fungicide), and fludioxonil (Maxim XL, fungicide). Pre-plant N-P-K was applied at a variable rate based on point soil samples and worked into the soil using conventional tillage. To prevent the germination of weeds, fields were sprayed just after planting with a pre-emergent herbicide containing atrazine and *S*-metolachlor. A post-emergent application of atrazine was administered approximately 40 days after planting. Sugarcane aphids (*Melanaphis sacchari*) were controlled with one or more applications of flupyradifurone (Sivanto Prime), and chlorantraniliprole (Prevathon) was administered in a single application to prevent corn earworm (*Helicoverpa zea*) and fall armyworm (*Spodoptera frugiperda*) infestation. The field trials were irrigated as required to prevent puzzling effects on genotypic performance due to maturity and varying degrees of drought tolerance.

### DArT genotyping

The MBL population was previously sequenced as described in Kumar et al. (2023). Briefly, DNA was extracted by Intertek (Alnarp, Sweden) from desiccated leaf punches collected from individual RILs and four parents at Florence, SC field site, with most plants at the grain filling stage. DNA samples were sent to Diversity Arrays Technology Pty Ltd. (Canberra, Australia (https://www.diversityarrays.com/); for Diversity Arrays Technology sequencing at low density (DArTseqLD). For DArTseqLD analysis, DNA wasdouble digested using *Pst*I and *Mse*I (Kilian et al., 2012), and amplified fragments were bulked and sequenced by the Hiseq2500 (Illumina^®^ Inc., San Diego, CA, United States). Resulting FASTQ data were processed using *fastp* (Chen et al., 2018) to remove barcodes and low-quality sequences before aligning reads with *BWA* (Li and Durbin, 2009). Variants were called using Genome Analysis Toolkit (*GATK*) (McKenna et al., 2010) best practices (DePristo et al., 2011; Van der Auwera et al., 2013). In brief, due to the nature of restriction digest, duplicates were not marked, but instead aligned reads went straight to base-quality recalibration. For recalibration, whole-genome sequencing data (Boatwright et al., 2022) with high quality (30x coverage) were used from the Sorghum Association Panel (Casa et al., 2008). The recalibrated BAM were then subjected to individual-sample variant calling to generate gVCFs before consolidating all gVCFs into a database for joint variant calling (Poplin et al., 2018). SNPs were hard filtered for quality (QD < 2.0, InbreedingCoeff < 0.0, QUAL < 30.0, SOR > 3.0, FS > 60.0, MQ < 40.0, MQRankSum < −12.5, and ReadPosRankSum < −8.0), missing data (50%), and minor allele frequency (>0.05) using both GATK and BCFtools, prior to GWAS. Beagle was used to impute missing genotype data in the VCF file assembled from GATK.

### Phenotypic data analysis

Pearson’s correlation coefficient matrix was generated using *metan* package and the *corr_plot* and *plot.corr_coef* functions were used for the visualization of correlation matrices for each trait in R software (R Foundation, 2020). Best linear unbiased predictions (BLUPs) values for each trait were calculated using random effect of the genotypes following *lme4* package in R (Bates et al., 2015). BLUPs values of each trait were used as response variables in GWAS analyses. The variance components for genotypes (*i.e.*, RILs) were estimated using the *lme4* package in R (Bates et al., 2015). All effects were treated as random. The *lme4*() function within this package optimized the linear mixed model using restricted maximum likelihood and was implemented to determine variance components for each random effect. Because there were two locations and 1 year, replicates and locations were used in the broad-sense heritability calculation in place of year along with interaction between genotype and location to estimate the variance caused by genotype x environment interaction as shown below.

$$H^{2}=\frac{\sigma_{G}^{2}}{\sigma_{G}^{2}+\frac{\sigma_{G\times R}^{2}}{R}+\frac{\sigma_{G\times L}^{2}}{L}+\frac{\sigma_{E}^{2}}{RL}}$$

where *G* is genotype, *R* is replicate, *L* is location, and *E* is error.

### Genome-wide association studies (GWAS)

GWAS were performed using a Memory-efficient, Visualization-enhanced, and Parallel-accelerated (*rMVP*) GWAS program (Yin et al., 2021) installed in the R programming language (R Foundation, 2020). The *rMVP* package was designed to process more efficiently the large GWAS datasets, quickly evaluate population structure, and implement parallel-accelerated association tests to dramatically improve computation time. Further, *rMVP* provides access to several of the most popular models including the mixed linear model (MLM; Zhang et al., 2010), and fixed and random model circulating probability unification (FarmCPU; Liu et al., 2016) model, which were both used for this study. The use of MLM permits a single-locus analysis, where individuals are included as random effects and the degree of correlation among individuals is determined using a kinship (K) matrix. The use of the MLM further provides shrinkage to the model such that potential false positives due to shared ancestry are no longer significant. An MLM can be described using Henderson’s matrix notation as follows:

Y=Xβ+Zu+e, (1)

where Y is the vector of observed phenotypes; β is an unknown vector containing fixed effects, including the genetic marker, population structure (Q), and the intercept; u is an unknown vector of random additive genetic effects for individuals/lines; X and Z are the known design matrices for fixed and random effects, respectively; and e is the unobserved vector of residuals. The u and e vectors are assumed to be normally distributed with zero mean and unit variance.

FarmCPU represents a multi-locus model that iteratively uses fixed and random effect models to generate sets of pseudo-quantitative trait nucleotides (QTNs) to use as covariates and control for false positives during analysis (Liu et al., 2016). FarmCPU provides benefits over traditional MLM as it performs a multi-locus analysis, may be efficiently computed, and removes confounding between kinship and the testing marker. By iterating a fixed effect model to identify significant pseudo-QTNs to use as covariates in a random effect model using a restricted kinship matrix like the SUPER algorithm (Wang et al., 2014a) to further refine the set of included covariates by maximizing the likelihood of the random effects model. Iterations cease when no change occurs in the estimated set of pseudo-QTNs. The significant marker trait associations, corresponding to putative SNPs for each trait were determined using Bonferroni-corrected *p*-value threshold 1.3e^−5^. This threshold was calculated using 0.05/*m*, with *m* being the number of markers at 3,751.

The linkage disequilibrium (LD) decay was estimated using PopLDdecay (Zhang et al., 2019) program within a 10 Mb window. LD decay was plotted for individual chromosomes as well as genome-wide using the custom R scripts (Boatwright et al., 2021), where the coefficient of determination (*r* ^*2*^) between markers located on each chromosome was measured to estimate the LD relationship between loci. The *r* ^*2*^ was plotted on the y-axis and physical distances (Mb) on the x-axis.

### Identification of allelic combinations independent of major dwarfing genes

GWAS were also performed for yield and yield component traits (GY, TGW, TN, and YPP) within a subset of RILs with genetic backgrounds fixed for the three dwarfing genes (*Dw1*, *Dw2,* and *Dw3*). For performing this analysis, we used two haplotypes (alternative alleles of each gene) based on the closest associated SNP with each gene (*Dw1*, *Dw2,* and *Dw3*) and performed GWAS analysis.

### Functional annotation of genes and QTL

Functional annotation was performed using reference genome BTx623 V3.1.1 to identify candidate genes associated with significant SNPs, which were identified through GWAS. Similarly, all the significant SNPs identified were used for validating the locations of MTAs based on the sorghum QTL atlas (Mace et al., 2019; aussorgm.org.au).

### Pleiotropic effects

Pleiotropic effects were assessed for all seven traits (PT, DTA, PH, GY, TGW, TN, and YPP) using the R package *mashr,* which uses a multivariate adaptive shrinkage approach to identify significant pleiotropic effects across the traits (Urbut et al., 2019). The estimated effect sizes and standard errors for every significant SNP marker in the MLM and FarmCPU for the above traits were filtered using a local false sign rate (LFSR) < 0.1 based on a condition-by-condition analysis using *mashr* in R (Stephens, 2017). The LFSR represents the probability of incorrectly assigning the direction of an effect. The LFSR provides a superior measure of significance over traditional multiple-testing corrections such as Bonferroni or False Discovery Rate (Benjamini and Hochberg, 1995) due to its robust estimation process (Stephens, 2017). A control set of estimated effects and standard errors were also randomly selected from the 3,751 markers to estimate the covariance between SNPs for each phenotype. Using this control set, a correlation matrix was estimated using *mashr* (Urbut et al., 2019) to control for any confounding effects arising from correlated traits. For testing pleiotropy across traits, canonical and data-driven covariance matrices were used. Posterior probabilities were calculated for each SNP by fitting a *mash* model on all tests. Bayes factors were extracted and plotted from *mash* results using the CDBN genomics R package (MacQueen et al., 2020). Variants exhibiting Bayes Factors greater than 10 were considered as demonstrating significant pleiotropic effects.

## Results

### Descriptive statistics, trait distribution, and heritability

The contrasting features among founders foreshadowed the wide range of phenotypic diversity of the MBL as summarized in Table 1. SC605 was an early flowering parent and reached anthesis at 57 days after planting, (DAP) while BTxARG-1 flowered significantly (16 days) later at 73 DAP. The RILs of MBL population showed a wide range of variation for flowering time. Early flowering line reached anthesis at 49 DAP at CU location compared to 50 DAP at FL location. Similarly, the late flowering line reached anthesis at 91 DAP at CU location compared to 89 DAP at FL location. The mean anthesis of the MBL RILs was 62 DAP at CU instead of 65 DAP at FL location (Supplementary S1). The range of PH among the founders was relatively narrow from 101 cm (BTx642) to 119 cm (SC630). BTxARG-1 had the highest grain yield followed by SC630 (kafir). Conversely, SC605 (guinea) was a poor yielder and displayed a high tillering capacity.

**TABLE 1:** Descriptive statistics and trait heritabilities of the MBL population.

Trait^a^	MBL parents				MBL population		H^2^ ^b^
Founder	SC630	SC605	BTx642	BTxARG-1	Mean	Range	
DTA	63 (5.0)	57 (4.2)	69.9 (4.3)	73.1 (8.0)	63.9	53.8–80.9	0.73
PH	119 (13)	115 (10.9)	101.3 (3.4)	113.4 (7.0)	130.7	69.4–201	0.84
GY	226 (48)	156 (37.7)	90 (27.5)	331 (59)	155.1	70.4–353	0.63
TGW	21.8 (2.4)	17.7 (0.9)	15.8 (3.9)	18.1 (1.4)	16.8	10.1–22.7	0.77
TN	16.3 (5.2)	24.8 (11)	7.1 (1.7)	16.8 (6.3)	18.3	13.3–26.5	0.34
YPP	15.5 (6.8)	7.0 (2.4)	12.9 (4.9)	22 (8.3)	9.4	5.3–20.4	0.60

^a^ DTA, days to anthesis; PH, plant height; GY, grain yield; TGW, 1000-grain weight, TN, tiller number per meter and YPP, yield per panicle.

^b^ Broad-sense heritability.

**FIGURE 1:**
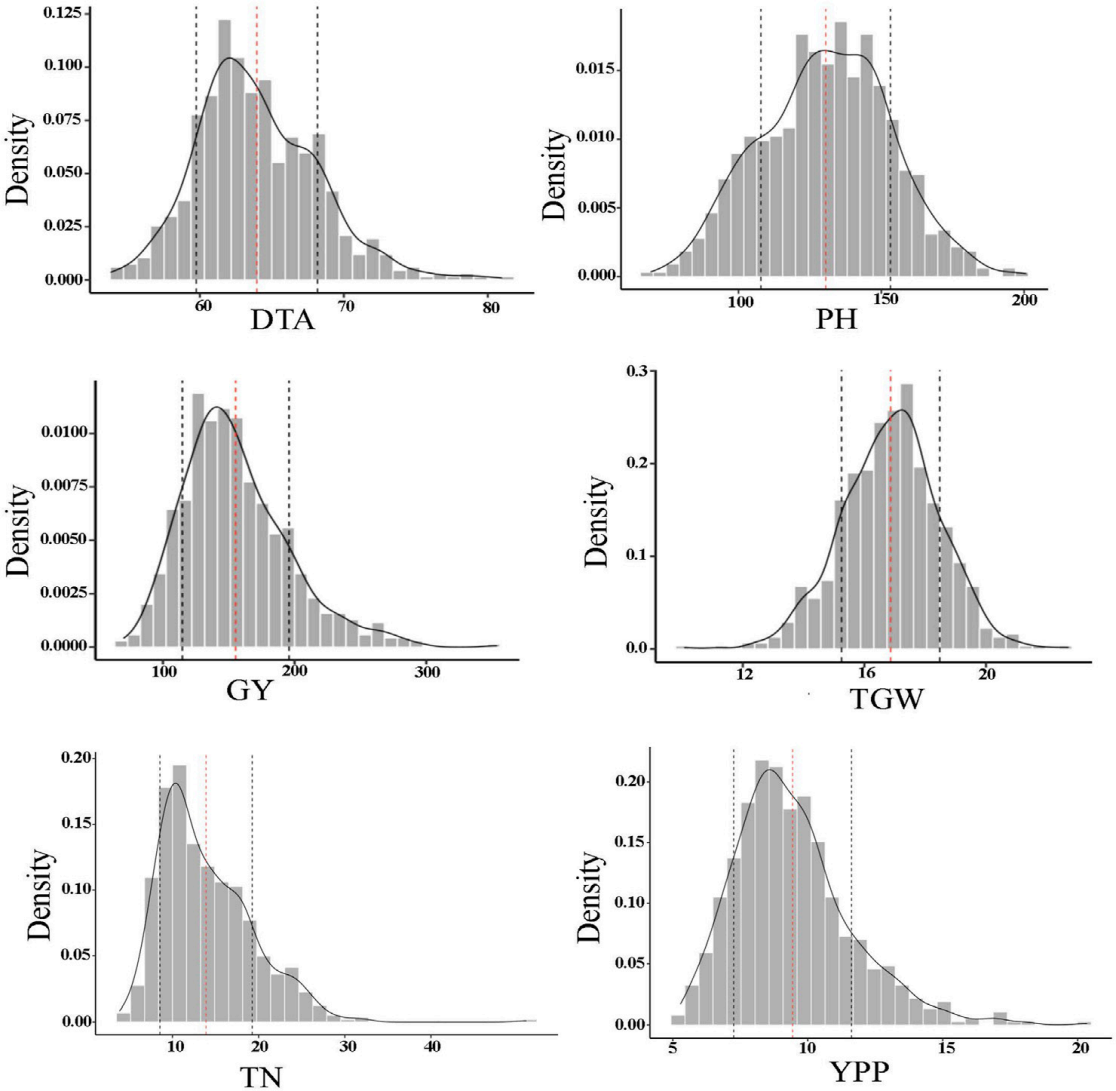
Frequency distribution of each phenotypic trait of the MBL population including DTA (days to anthesis), PH (plant height), GY (grain yield), TGW (1000-grain weight), TN (tiller number per meter), and YPP (yield per panicle).

In addition, the MBL population showed transgressive segregation for the majority of the traits (Table 1). The range of PH was wide in the RILs (69–201 cm) and an average of the RILs was significantly higher at 130.7 cm compared to parental lines. The results of variance component analysis demonstrated that variances due to genotype (line), genotype x location, and blocks within replication and location had significant contributions to total phenotypic variance for each trait (DTA, PH, GY, TGW, TN, and YPP) of the genotype (Supplementary Table S1). Environmental effects on phenotypic trait values were largely from blocks within replication and variance due to location for all the traits except GY, however, it showed a highly significant effect due to genotype x location. The MBL population showed wide and continuous distribution for the six quantitative traits (DTA, PH, GY, TGW, TN, and YPP) as expected (Figure 1). Estimates of broad sense heritability were lowest for TN (0.34) and highest for PH (0.84) (Table 1). Heritability was on the higher side for TGW (0.77) while moderate for GY (0.63) and YPP (0.60). Since, PT was an ordinal trait thus excluded from this analysis.

### Relationships among phenotypic traits

Panicle type (PT) did not show any relationship with DTA, TGW, and TN, while it showed poor positive correlations with PH (r = 23), GY (r = 0.12), and YPP (r = 0.11) (Table 2). DTA was negatively correlated with TN (r = −0.40) and TGW (r = −0.17), but no significant relationship was found with PH or GY. PH showed a significant and positive correlation with GY (r = 0.50) and yield component traits (TGW, TN, and YPP). GY had a strong positive correlation with TGW (r = 0.44) and YPP (r = 0.78) while moderate with TN (r = 0.31). TGW was positively correlated with TN and YPP. TN displayed a negative relationship with YPP. Overall, strong and positive correlations were observed among PH, GY, TGW, and YPP across the locations (Supplementary Figure S1).

**TABLE 2:** Estimates of Pearson’s correlation coefficients among phenotypic traits of the MBL population.

Trait^a^	PT	DTA	PH	GY	TGW	TN	YPP
PT	1						
DTA	0.01	1					
PH	0.23***	0.03	1				
GY	0.12**	0.07	0.50***	1			
TGW	0.01	−0.17**	0.42***	0.44***	1		
TN	−0.01	−0.40***	0.24***	0.31***	0.31***	1	
YPP	0.11**	0.26***	0.41***	0.78***	0.26***	−0.22***	1

^a^ ^,^ PT, panicle type; DTA, days to anthesis; PH, plant height; GY, grain yield; TGW, 1000-grain weight, TN, tiller number per meter; and YPP, yield per panicle. * Significance at the 0.05 probability level. **Significance at the 0.01 probability level; ***Significance at the 0.001 probability level.

### GWAS for quantitative traits

**FIGURE 2:**
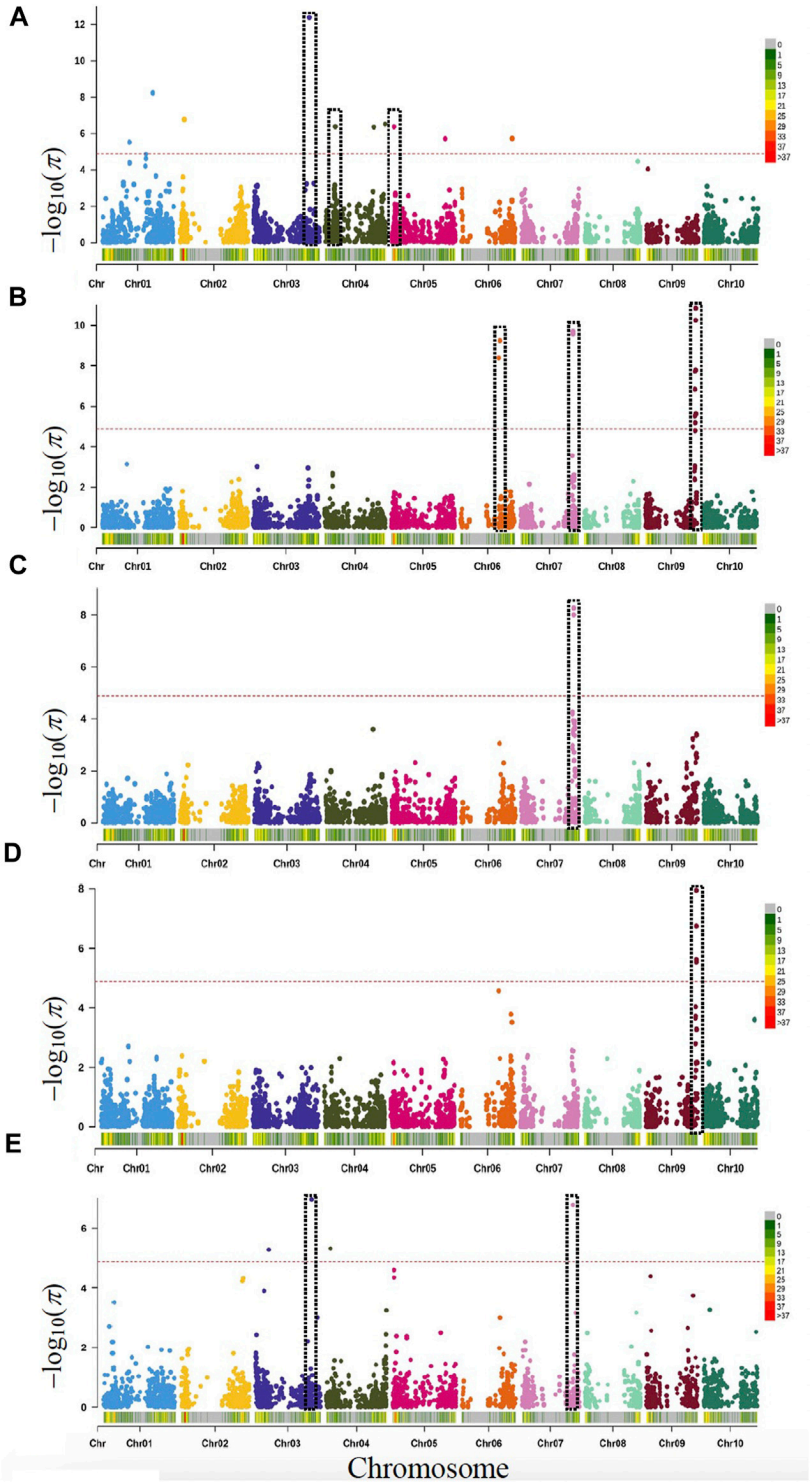
Manhattan plots based on *rMVP*-GWAS program using MBL population with highlighted genes or loci identified for various traits. Vertical dotted bars show genes and loci related to **(A)** days to anthesis (DTA: Chr3, Chr4 & Chr5); **(B)** Plant height or dwarfing genes (*Dw1:* Chr9, *Dw2:* Chr6, and *Dw3:* Chr7); **(C)** Grain yield and *Dw3* (Chr7); **(D)** 1000-grain weight and *Dw1* (Chr9); **(E)** Yield per plant and *Dw3* (Chr3 and Chr7). The -log_10_ (*p*) values (*y*-axis) are plotted against the position on each chromosome (*x*-axis). Each solid circle represents a SNP, and the red dashed line represents the Bonferroni-corrected threshold (*p* ≤ *0.05*).

GWAS were performed using MLM as a single-locus and the FarmCPU as a multi-locus model to predict genotype-by-phenotype associations. BLUPs of phenotypes were used to minimize errors across the multi-environment data more efficiently to identify QTL or MTAs in GWAS or QTL mapping studies. The MTAs were defined based on the rate of average LD decay observed in our population. In MBL, genome-wide LD fell around 2.5 Mb, therefore any two or more loci detected apart from 2.5 Mb distances were considered different MTA. Altogether, GWAS identified 70 significant associations (52 MTAs) for seven targeted traits. These 52 MTAs were distributed on 32 genomic regions (*i.e.*, linkage blocks) across the 10 chromosomes of sorghum, except chromosome 8 (Figure 2, Supplementary Table S2, and Supplementary Figure S2). Of these 70 associations, 46 significant SNPs were identified by FarmCPU alone, only 10 were identified by MLM and 14 associations were commonly identified by both models. Several significant MTAs across the traits were corroborated with previous studies conducted for the corresponding traits while some of them were novel associations.

### Panicle type

The FarmCPU model identified three significant MTAs for PT, which were located on Chr1 (∼24 Mb), Chr2 (∼58 Mb), and Chr6 (∼43 Mb). No significant association was identified by MLM. Of these three associations, a sole SNP (Chr6:43,244,873) overlapped with PH and GY. This genomic region at ∼43 Mb was within 0.50 Mb of the dwarfing gene *Dw2* (Sobic.006G067700).

### Agronomic traits

In total, 12 MTAs were significantly associated with DTA, which were located on seven different chromosomes (Chr1, Chr2, Chr3, Chr4, Chr5, Chr6, and Chr10). Among these, nine MTAs were uniquely detected by FarmCPU, whereas two MTAs were detected using MLM, and a single SNP was commonly detected by both models. Two significant MTAs between DTA and PH overlapped, which were located on Chr3 (Chr3:62,831,536) and on Chr5 (Chr5:2,588,266) (Figure 2; Supplementary Table S2, and Supplementary Figure S2).

In total, 10 significant MTAs (21 SNPs) were identified for PH, which were located on seven different chromosomes (Chr2, Chr3, Chr4, Chr6, Chr7, Chr9, and Chr10). A single MTA carrying 10 significant SNPs was associated with Chr9, which spanned a ∼1.3 Mb region (∼57 Mb) within a LD block. Out of 10 significant SNPs, one SNP was positioned on Chr9 (Chr9:57,030,394) near *Dw1* (Sobic.009G229800). Another MTA was detected on Chr6 (Chr6:43,244,873; Chr6:44,567,620) in the same LD block being ∼0.43 Mb to *Dw2* (Sobic.006G067700). For PH, a significant MTA was identified on Chr7 (Chr7:59,606,838), in proximity (0.21 Mb) to *Dw3* (Sobic.007G163800). In addition to these PH loci, seven additional MTAs that passed the significant threshold were identified including three on Chr3, two on Chr2, and one each on Chr4 and Chr10. Interestingly, a common MTA was detected on Chr3 (Chr3:62,831,536) around ∼62 Mb for both the agronomic (DTA and PH) traits (Supplementary Table S2, and Supplementary Figure S2).

### Yield component traits

Altogether, GWAS identified 27 significant MTAs for grain yield and its components (GY, TGW, TN, and YPP) including several shared associations among the traits (Figure 2 and Supplementary Table S2, and Supplementary Figure S2). Four MTAs (five SNPs) were associated with GY, which were located on Chr6 (∼43 Mb), Chr7 (∼59 Mb), and Chr9 (∼57 Mb). All three significant loci overlapped with three major dwarfing genes (*Dw1*, *Dw2*, and *Dw3*). Of these five SNPs, three were independently detected by FarmCPU and a sole SNP (Chr7:59,591,981) was detected by MLM, whereas only one SNP (Chr7:59,606,838) detected by both models (FarmCPU and MLM) within the same LD block. All four MTAs showed shared associations with all traits (PT, PH, GY, TGW, TN, and YPP) except DTA.

Five MTAs (eight SNPs) were identified for TGW in total, which were located on five different chromosomes (Chr1, Chr2, Chr3, Chr9, and Chr10). Of these five associations, one MTA (four SNPs) was significantly associated with a locus on Chr9 at ∼57 Mb that spanned a genomic region (0.75 Mb). This genomic region showed significant association with multiple traits including TGW (PH, GY, and TN). In total, GWAS identified 12 SNPs (10 MTAs) significantly associated with TN, which were located on seven different chromosomes (Chr1, Chr3, Chr4, Chr5, Chr7, Chr9, and Chr10). All the significant associations were detected by FarmCPU. Three genomic regions, each located on Chr5 (∼25 Mb), Chr7 (∼59 Mb), and Chr9 (∼57 Mb) showed common associations with multiple traits (TN, DTA, PH, GY, and TGW).

Application of FarmCPU to YPP resulted in the identification of eight MTAs, which were located on six chromosomes (Chr2, Chr3, Chr4, Chr5, Chr7, and Chr9). Of these eight MTAs, a locus on Chr2 (∼59 Mb) showed a significant association with PH. Similarly, another significant MTA on Chr7 (∼57 Mb) showed association with multiple traits including YPP, PH, and GY (Figure 2 and Supplementary Table S2, and Supplementary Figure S2).

### Pleiotropic QTL and high-resolution power of MBL

All the significant SNPs identified through GWAS following two models (FarmCPU and MLM) were used to assess pleiotropic effects for all the seven traits using the *mashr* program. A total of 15 significant MTAs (29 SNPs) were detected with pleiotropic effects on multiple traits (Table 3; Supplementary Table S3, and Supplementary Figure S3). These MTAs were distributed on all the chromosomes of sorghum except Chr2 and Chr8. In this analysis, PH showed nine shared associations with six additional traits (PT, DTA, GY, TGW, TN, and YPP) involving five genomic regions located on five different chromosomes (Chr2, Chr3, Chr6, Chr7, and Chr9). GY showed four shared associations with five yield components (PT, PH, TGW, TN, and YPP) that involved three genomic regions located on Chr6, Chr7, and Chr9. Similarly, TGW also showed four shared associations with three traits (PH, GY, and TN) involving a repeatedly detected locus on Chr9 (∼57 Mb). TN showed three shared associations with four traits (DTA, PH, GY, and TGW) involving three chromosomes (Chr5, Chr7, and Chr9). YPP showed two shared associations with two traits (PH, and GY) involving two genomic regions located on Chr2 (∼59 Mb) and Chr7 (∼59 Mb) (Table 3; Supplementary Table S3). Overall, the most common genomic regions located on Chr7 (∼59 Mb) and Chr9 (∼57 Mb) were significantly associated with multiple traits (PH, GY, TGW, and YPP).

**TABLE 3:** A summary of pleiotropic QTL identified for various traits using MBL population.

Trait^a^	Chromosome	Position (bp)	Effect	SE	Probability	Pleiotropy
PT	Chr06	43,244,873	−0.20	0.04	2.00E-06	PT + PH + GY
DTA	Chr03	62,831,536	−1.16	0.17	4.27E-13	DTA + PH
	Chr05	2,588,266	−0.74	0.18	4.23E-07	DTA + TN
PH	Chr02	59,202,168	4.26	1.02	1.10E-06	PH + YPP
	Chr03	62,831,536	−4.20	0.79	6.03E-10	DTA + PH
	Chr06	43,244,873	−4.46	1.01	1.24E-05	PT + PH + GY
	Chr07	59,591,981	9.06	0.67	4.79E-42	PH + GY + TN
	Chr07	59,606,838	8.72	0.66	8.58E-42	PH + GY + YPP
	Chr09	57,030,394	7.42	0.96	4.37E-14	PH + TGW + TN
	Chr09	57,074,147	9.88	1.48	5.6E-11	PH + TGW
	Chr09	57,074,148	9.90	1.49	5.6E-11	PH + TGW
	Chr09	57,106,095	8.07	0.97	5.25E-16	PH + GY + TGW
GY	Chr06	43,244,873	−8.95	1.90	7.30E-07	PT + PH + GY
	Chr07	59,591,981	12.72	2.19	1.0E-08	PH + GY + TN
	Chr07	59,606,838	12.39	2.10	5.5E-09	PH + GY + YPP
	Chr09	57,106,095	8.11	1.64	1.97E-07	PH + GY + TGW
TGW	Chr09	57,030,394	0.73	0.06	3.98E-30	PH + TGW + TN
	Chr09	57,074,147	0.51	0.11	2.9E-06	PH + TGW
	Chr09	57,074,148	0.52	0.11	2.4E-06	PH + TGW
	Chr09	57,106,095	0.58	0.11	1.8E-07	PH + GY + TGW
TN	Chr05	2,588,266	0.29	0.09	9.52E-06	DTA + TN
	Chr07	59,591,981	0.35	0.07	8.44E-07	PH + GY + TN
	Chr09	57,030,394	0.34	0.07	1.29E-06	PH + TGW + TN
YPP	Chr02	59,202,168	0.43	0.10	7.91E-06	PH + YPP
	Chr07	59,606,838	0.50	0.09	2.49E-08	PH + GY + YPP

^a^ PT, panicle type; DTA, days to anthesis; PH, plant height; GY, grain yield; TGW, 1000-grain weight.

TN, tiller number per meter; and YPP, yield per panicle.

### Identification of allelic combinations independent of major dwarfing genes

GWAS were also performed for yield related traits within a subset of RILs with genetic background fixed for the three dwarfing genes (*Dw1*, *Dw2,* and *Dw3*) that were segregating in the MBL population. Using this approach, two additional MTAs were identified on two different chromosomes (Chr1 and Chr7) that were all independent from *Dw1*, *Dw2*, and *Dw3* (Supplementary Table S4). Both these MTAs were novel genomic loci, each was located on Chr1 (∼11 Mb) and Chr7 (∼7.5 Mb). A total of eight haplotypes were formed based on the three major dwarfing genes (*Dw1*, *Dw2,* and *Dw3*) identified in the four founders of this population (Table 4; Supplementary Table S4).

**TABLE 4:** Average phenotypic trait values of all possible SNP haplotypes based on three major dwarfing genes in sorghum (*Dw1, Dw2,* and *Dw3*) along with four founders of the MBL and the top selected lines.

Line ID	Gene-haplotype	SNP haplotype	PH	GY	TGW	TN	YPP
SC630	*Dw1dw2dw3*	*GAG*	117	179	21	15	13
BTxARG-1	*dw1Dw2dw3*	*C-G*	108	169	16	16	12
SC605	*dw1dw2Dw3*	*CAA*	118	126	17	19	7
BTx642	*dw1dw2dw3*	*CAG*	99	71	14	13	8
*n* = 82	*Dw1dw2dw3*	*GAG*	122	124	17	19	7
*n* = 68	*dw1Dw2dw3*	*C-G*	113	139	16	16	9
*n* = 93	*dw1dw2Dw3*	*CAA*	113	135	16	18	8
*n* = 43	*dw1dw2dw3*	*CAG*	96	94	14	17	6
*n* = 36	*Dw1Dw2Dw3*	*G-A*	145	160	18	20	9
*n* = 13	*Dw1Dw2dw3*	*G-G*	121	111	17	17	7
*n* = 167	*dw1Dw2Dw3*	*C-A*	147	196	17	18	12
*n* = 120	*Dw1dw2Dw3*	*GAA*	145	165	18	22	9
MBL0911	*dw1Dw2Dw3*	*C-A*	154	377	19	25	18
MBL0963	*dw1Dw2Dw3*	*C-A*	128	362	15	19	20
MBL0918	*dw1Dw2dw3*	*C-G*	151	331	18	18	21
MBL0987	*dw1dw2Dw3*	*CAA*	136	324	15	19	18
MBL0088	*Dw1dw2Dw3*	*GAA*	156	313	18	19	17

PH, plant height; GY, grain yield; TGW, 1000-grain weight, TN, tiller number per meter; and YPP, yield per panicle. The meaning of italic values are Dw1, Dw2, and Dw3 denoted as dwarfing1, dwarfing2, and dwarfing3, respectively.

## Discussion

Sorghum is predominantly grown as a hybrid crop in the United States. Currently, cytoplasmic male sterility (CMS) is a popular method of hybrid seed production. The CMS system requires three lines (A-, B-, and R) for cross-fertilization (Xin et al., 2021). As indicated by the name MBL (MAGIC B-line), this population comprises a unique source of B- or maintainer progeny lines, which are used to develop new female (*i.e.,* seed) parents (Rooney, 2004). This population was created to increase genetic diversity in the narrower female sorghum gene pool and facilitates the identification of new genetic variants for future female parent development. Founders of the MBL population were purposely chosen for their ability to maintain sterility to A_1_ cytoplasm while capturing genetic diversity across the primary botanical races (kafir, guinea, durra, and caudatum). As a result, the high phenotypic variance was observed for all the phenotypic traits (PT, DTA, PH, GY, TGW, TN, and YPP) in the MBL population. In this population, our major focus was to identify desirable allelic combinations and genetic loci associated with yield influencing traits that can be used for sorghum yield improvement in the future.

### Relationship among traits and pleiotropic effects

Interestingly, we observed several colocalized or overlapped significant MTAs between four yield influencing traits (PH, GY, TGW, and YPP), which was reflected by the relationship among these traits (Table 2; Supplementary Figure S3A,B). The overall phenotypic pair-wise correlations among the four traits (PH, GY, TGW, and YPP) were significantly positive, except TN and YPP, which showed a poor and negative correlation. We identified 15 MTAs commonly associated (shared associations) with multiple traits through pleiotropic analysis particularly between positively correlated traits (PH, GY, TGW, and YPP). These correlated traits can be simultaneously improved by selecting a single trait via indirect phenotypic selection. Conversely, TN did not show any correlation with YPP and resulted in no colocalization of QTL. Similarly, PT, DTA, and PH exhibited no significant correlation between them or with the above traits. Therefore, colocalization was rarely observed between these traits (PT, DTA, and PH), which suggests that they are largely under independent genetic control in this population. QTL mapping studies made similar observations in context of the colocalization of QTL between multiple traits in sorghum (Mackay et al., 2009; Boyles et al., 2017b; Olatoye et al., 2020).

### Panicle type

Panicle type (*i.e*., morphology) is an important trait that facilitated racial classification in sorghum. Panicle morphology also influences traits related to crop adaptation such as grain maturity, grain yield, and grain size (Brown et al., 2006; Hmon et al., 2013). However, the genetic architecture of panicle morphology is not completely understood in sorghum, only a few genes have been characterized so far (Morris et al., 2013; Hmon et al., 2014; Wang et al., 2021). The four founders of the MBL belong to four different races and each founder has different panicle morphology. SC630 (kafir) has a compact panicle in contrast to the open panicle type founder (SC605; guinea), whereas BTx642 (durra) and BTxARG-1 (caudatum) are semi-open and semi-compact types, respectively. We identified three significant MTAs for PT, located on three different chromosomes (Chr1, Chr2, and Chr6). A candidate gene (Sobic.006G067700) was detected within the 0.50 Mb region of the dwarfing gene (*Dw2*), which has been mapped earlier in sorghum (Klein et al., 2008; Hilley et al., 2017). The same locus overlapped with panicle length (Zhang et al., 2015). Other genetic loci associated with panicle length have been previously reported in sorghum on Chr2 (Shehzad and Okuno, 2015), and Chr6 (Zhou et al., 2019) in the overlapping regions. The association found on Chr1 is novel.

### Agronomic traits

The MBL population demonstrated wide continuous distributions for DTA and PH, which indicates the quantitative nature of these traits (Figure 1). GWAS identified the most significant associations (12 MTAs) for DTA, which were located on seven different chromosomes. This is not surprising, because the genetic loci associated with DTA have been reported earlier on all the ten chromosomes of sorghum (Boatwright et al., 2022). Of the 12 significant associations of DTA, a locus (QDTFL1.53) on Chr1 (∼57 Mb) overlapped in previous studies (Mace et al., 2013; Burks et al., 2015). Similarly, two additional loci, one on Chr4 (QDTFL4.18) and another on Chr6 (QDTFL6.56) overlapped in earlier studies by Mace et al. (2013), and Sangma (2013), respectively. The rest of the genetic loci identified for DTA were novel.

As we know, three major dwarfing genes have been previously reported in sorghum on different chromosomes, such as *Dw1* (Sobic.009G229800) on Chr9 (Brown et al., 2008; Klein et al., 2008), *Dw2* (Sobic.006G067700) on Chr6 (Wang et al., 2014b; Higgins et al., 2014; Burrell et al., 2015), and *Dw3* (Sobic.007G163800) on Chr7 (Morris et al., 2013; Girma et al., 2019; Boatwright et al., 2022). For PH, 10 MTAs were identified, which were located on seven different chromosomes (Chr2, Chr3, Chr4, Chr6, Chr7, Chr9, and Chr10). In this study, the most significant SNPs were located on three chromosomes (Chr6, Chr7, and Chr9), which overlapped with major dwarfing genes/QTLs known in sorghum such as Chr6 (*Dw2*), Chr7 (*Dw3*), and Chr9 (*Dw1*). In addition, three common genetic loci associated with PH on the three chromosomes (Chr1, Chr7, and Chr9), have been mapped earlier by Boyles et al. (2017b) using two biparental populations sharing founders (BTx642 and BTxARG-1) of the MBL population. Additional genetic loci identified in this study were also reported earlier studies in the overlapping regions, each located on the Chr3 (QHGHT3.3) (Hart et al., 2001; Feltus et al., 2006; Wang et al., 2014b), Chr4 (Wang et al., 2014b), Chr9 (QHGHT9.30) (Felderhoff et al., 2012; Takai et al., 2012; Wang et al., 2014b), and Chr10 (Parra-Londono et al., 2018) using biparental population and diverse panel of sorghum.

### Yield component traits

GY is a complex trait determined by many yield components like grain weight, tiller number per unit area, and grain yield per panicle (Boyles et al., 2016; Boyles et al., 2017b; reviewed in Baye et al., 2022). Since GY and its component traits are polygenic in nature, they exhibit continuous phenotypic distributions (Figure 1). For GY, three of the four MTAs detected each on Chr6 (∼43 Mb), Chr7 (∼59 Mb), and Chr9 (∼57 Mb) were overlapped with three major dwarfing genes namely *Dw2*, *Dw3,* and *Dw1*, respectively. These observations indicated the potential influence of dwarfing genes on other traits. Genetic loci associated with GY have been overlapped on Chr6 (Leiser et al., 2014), Chr7 (Guindo et al., 2019), and Chr9 in sorghum (Sabadin et al., 2012; Reddy et al., 2013; Reddy et al., 2014; Boyles et al., 2017b). A novel significant MTA was detected on Chr9 (∼52 Mb). Interestingly, these three genomic regions exhibited significant shared associations with all the phenotypic traits attempted in this study, except DTA.

Grain weight is one of the major yield components and highly heritable traits (Boyles et al., 2016; 2017b; reviewed in Baye et al., 2022). In total, five significant MTAs were identified for TGW, which were located on five different chromosomes (Chr1, Chr2, Chr3, Chr9, and Chr10). Of these five, three MTAs overlapped on Chr1 and Chr2 (Boyles et al., 2017b; Patil et al., 2019), and Chr3 (Sabadin et al., 2012). Another locus for TGW has been mapped in the same region on Chr9 (∼57 Mb) in previous studies (Reddy et al., 2013; Boyles et al., 2016). The above locus also showed a shared association with multiple traits (PH, GY, and TN). However, a locus associated with TGW on Chr 10 (∼35 Mb) was identified for the first time in our study. In continuation of grain yield components, TN is also an important trait, that determines the grain yield by increasing the number of tillers or panicles per unit area in sorghum. For TN, 10 significant MTAs were identified, which were located on seven different chromosomes (Supplementary Table S2). Of these 10 associations, three MTAs were previously mapped using multiple populations in the overlapping regions on Chr1, and Chr3 (Alam et al., 2014; Kong et al., 2014), and Chr4 (Alam et al., 2014). A genomic region associated with multiple traits (TN, GY, and YPP) was identified on Chr9 (∼57 Mb), which was reported earlier for tiller numbers (Feltus et al., 2006; Zhang et al., 2015). However, another locus associated with the same set of multiple traits (TN, GY, and YPP) on Chr7 (∼59 Mb) was identified in this study, which is a novel association for tiller numbers. YPP directly influences the overall grain yield in sorghum by several factors such as high grain weight and grain number per panicle. Altogether, eight significant MTAs were identified for YPP, which were located on six chromosomes (Chr2, Chr3, Chr4, Chr5, Chr7, and Chr9). A locus was identified on Chr2 (∼59 Mb) that was previously mapped in the overlapping region (Shehzad and Okuno, 2015). Two genetic loci each on Chr7 (∼59 Mb) and Chr9 (∼57 Mb) were identified for YPP. Both the common genomic regions associated with grain yield components located on Chr7 and Chr9 have been previously mapped in the same region (Boyles et al., 2017b) using two biparental RIL populations sharing founders (BTx642 and BTxARG-1) of the MBL population.

### High resolution power of MBL

High resolution mapping is a critical step for the identification of novel genes and narrows down the genetic distance between the candidate genes associated with complex traits in crop plants (Huang et al., 2015; Scott et al., 2020; Kumar et al., 2023). As we reported earlier (Kumar et al., 2023), the genome-wide LD decayed much more quickly in the MBL compared to a biparental population (Supplementary Figure S4), which is consistent with previous comparisons between biparental and MBL populations (Boyles et al., 2017a). Based on the previous mapping studies, the MAGIC populations are more efficient to narrow down the genomic regions compared to biparental populations (Huang et al., 2012; Kumar et al., 2023). Here, we compared our results with an earlier QTL mapping study performed by Boyles et al. (2017b), where two different biparental RIL mapping populations were used for QTL discoveries sharing two of the four founders (BTxARG1 and BTx642) of the MBL. Comparing the common genomic regions identified on three chromosomes (Chr2, Chr7, and Chr9) in both the studies for the same set of quantitative traits (PH, TGW, and YPP), MBL placed all the common QTL in narrow genomic regions as compared to biparental populations (Boyles et al., 2017b). The potential of the MAGIC populations in facilitating high resolution mapping have been reported earlier in crops with even more complex genomes like wheat (Huang et al., 2012; Stadlmeier et al., 2018) and cotton (Islam et al., 2016).

### Significance of MBL in sorghum improvement

Despite MBL parents all having short stature (Table 4), three of the four founders displayed dominance at a unique locus SC630 (*Dw1*), BTxARG1 (*Dw2*), and SC605 (*Dw3*). As a result, all eight gene combinations were present across MBL progeny, which led to significant height variation. Because plant height confounds grain yield and related traits, these dwarfing genes tended to dominate explained phenotypic variance and thus masked the effects of other genetic variants segregating in the population. To identify novel alleles associated with yield related traits, GWAS were performed using a subset of RILs that contained matching plant height gene combinations. Using this approach, seven significant genomic loci were identified on five different chromosomes (Chr1, Chr3, Chr4, Chr6, and Chr7) for yield influencing traits. These genomic regions overlapped with multiple yield component traits (GY, TGW, TN, and YPP). Of these seven, the two significant loci (Chr1 and Chr7) identified appear to be novel allelic combinations. This MBL population was designed to identify and recombine useful genetic variation present in diverse maintainer (B-line) germplasm. Desirable allelic combinations were indeed elucidated based on phenotypic comparisons of all the MBL lines for yield related traits. Considering plant height allelic combinations, five best-performing MBL progeny lines were selected (Table 4). These favorable recombinants should be hybridized with elite pollinator parents to test for general combining abilities to help determine their value in sorghum hybrid development. To reinforce favorable recombination, various MBL progeny exhibited transgressive segregation for all the phenotypic traits (DTA, PH, GY, TGW, TN, and YPP), which indicates the distribution of positive and negative alleles in the founders. Such segregation and allele shuffling provide opportunities for trait improvement through pyramiding desirable alleles from selected progeny lines.

## Conclusion

The MBL is a structured multi-parent population that encompasses a rich source of genetic variation for the seed parent gene pool (also referred to as the female, A/B-line, or maintainer pools). Seed parent genetic diversity within the A/B/R CMS system is limited due to the majority of sorghum genotypes being partial or full fertility restorers based on one or more nuclear restoration genes. Here, allelic variants segregating among four diverse founder lines were mined for association with complex, quantitative traits using GWAS. Specific traits included PT, agronomic (DTA and PH), grain yield (GY), and yield components (TGW, TN, and YPP). GWAS identified 52 MTAs located on all the chromosomes of sorghum except Chr8, representing both novel and previously identified genetic loci for the above traits in narrow genetic regions. In addition, 15 significant MTAs were identified with pleiotropic effects involving grain yield and yield influencing traits. Desirable allelic combinations were identified based on plant height haplotypes associated with plant height genes: *Dw1* (Chr9), *Dw2* (Chr6), and *Dw3* (Chr7). Additionally, two novel MTAs each on Chr1, and Chr7, were identified for grain yield and yield component traits when dwarfing genes (*Dw1*, *Dw2,* and *Dw3*) were fixed in a subset of MBL progeny. Favorable alleles at these loci can be leveraged for continued improvement of sorghum seed parent productivity and performance. Direct selection on these pleiotropic loci, in the absence of linkage between favorable and deleterious alleles, can be used to simultaneously improve correlated traits. Finally, at least five best-performing RILs were selected for validation of grain yield and yield related traits associated with dwarfing genes allelic combinations segregating in the MBL population. The favorable progeny lines can serve as valuable germplasm in sorghum hybrid breeding programs.

## Data Availability

The datasets presented in this study can be found in online repositories. The names of the repository/repositories and accession number(s) can be found in the article/Supplementary Material.

## Abbreviations

BLUPs, best linear unbiased predictions; MAGIC, multi-parent advanced generation inter-cross; MBL, MAGIC B-line; QTL, quantitative trait locus; PT, panicle type, DTA, days to anthesis; PH, plant height; GY, grain yield; TGW, 1000-grain weight; TN, tiller number per meter; YPP, yield per panicle.

## References

[B1] Alam M. M. Mace E. S. van Oosterom E. J. Cruickshank A. Hunt C. H. Hammer G. L. (2014). QTL analysis in multiple sorghum populations facilitates the dissection of the genetic and physiological control of tillering. Theor. Appl. Genet. 127, 2253–2266. 10.1007/s00122-014-2377-9 25163934

[B2] Ayana A. Bekele E. (1998). Geographical patterns of morphological variation in sorghum (*Sorghum bicolor* (L.) Moench) germplasm from Ethiopia and Eritrea: qualitative characters. Hereditas 129, 195–205. 10.1111/j.1601-5223.1998.t01-1-00195.x

[B3] Bandillo N. Raghavan C. Muyco P. A. Sevilla M. A. Lobina I. T. Dilla-Ermita C. J. (2013). Multi-parent advanced generation inter-cross (MAGIC) populations in rice: progress and potential for genetics research and breeding. Rice 6 (1), 11–15. 10.1186/1939-8433-6-11 24280183 PMC4883706

[B4] Barnaud A. Trigueros G. McKey D. Joly H. I. (2008). High outcrossing rates in fields with mixed sorghum landraces: how are landraces maintained? Heredity 101 (5), 445–452. 10.1038/hdy.2008.77 18685567

[B5] Bates D. Kliegl R. Vasishth S. Baayen H. (2015). Parsimonious mixed models. arXiv preprint arXiv:1506.04967.

[B6] Baye W. Xie Q. Xie P. (2022). Genetic architecture of grain yield-related traits in sorghum and maize. Int. J. Mol. Sci. 23 (5), 2405. 10.3390/ijms23052405 35269548 PMC8909957

[B7] Benjamini Y. Hochberg Y. (1995). Controlling the false discovery rate: A practical and powerful approach to multiple testing. J. R. Stat. Soc. 57 (1), 289–300. 10.1111/j.2517-6161.1995.tb02031.x

[B8] Boatwright J. L. Brenton Z. W. Boyles R. E. Sapkota S. Myers M. T. Jordan K. E. (2021). Genetic characterization of a Sorghum bicolor multiparent mapping population emphasizing carbon-partitioning dynamics. G3 Genes, Genomes, Genet. 11 (4), jkab060. 10.1093/g3journal/jkab060 PMC8759819 33681979

[B9] Boatwright J. L. Sapkota S. Jin H. Schnable J. C. Brenton Z. Boyles R. (2022). Sorghum Association Panel whole‐genome sequencing establishes cornerstone resource for dissecting genomic diversity. Plant J. 111 (3), 888–904. 10.1111/tpj.15853 35653240 PMC9544330

[B10] Boyles R. E. Cooper E. A. Myers M. T. Brenton Z. Rauh B. L. Morris G. P. (2016). Genome‐wide association studies of grain yield components in diverse sorghum germplasm. Plant Genome 9 (2), plantgenome2015.09.0091. 10.3835/plantgenome2015.09.0091 27898823

[B11] Boyles R. E. Pfeiffer B. K. Cooper E. A. Rauh B. L. Zielinski K. J. Myers M. T. (2017a). Genetic dissection of sorghum grain quality traits using diverse and segregating populations. Theor. Appl. Genet. 130, 697–716. 10.1007/s00122-016-2844-6 28028582 PMC5360839

[B12] Boyles R. E. Pfeiffer B. K. Cooper E. A. Zielinski K. J. Myers M. T. Rooney W. L. (2017b). Quantitative trait loci mapping of agronomic and yield traits in two grain sorghum biparental families. Crop Sci. 57 (5), 2443–2456. 10.2135/cropsci2016.12.0988

[B13] Brown P. J. Klein P. E. Bortiri E. Acharya C. B. Rooney W. L. Kresovich S. (2006). Inheritance of inflorescence architecture in sorghum. Theor. Appl. Genet. 113, 931–942. 10.1007/s00122-006-0352-9 16847662

[B14] Brown P. J. Rooney W. L. Franks C. Kresovich S. (2008). Efficient mapping of plant height quantitative trait loci in a sorghum association population with introgressed dwarfing genes. Genetics 180, 629–637. 10.1534/genetics.108.092239 18757942 PMC2535712

[B15] Burks S. V. Cowgill B. Hoffman M. Housman M. (2015). The value of hiring through employee referrals. Q. J. Econ. 130 (2), 805–839. 10.1093/qje/qjv010

[B16] Burrell A. M. Sharma A. Patil N. Y. Collins S. D. Anderson W. F. Rooney W. L. (2015). Sequencing of an anthracnose‐resistant sorghum genotype and mapping of a major QTL reveal strong candidate genes for anthracnose resistance. Crop Sci. 55 (2), 790–799. 10.2135/cropsci2014.06.0430

[B17] Casa A. M. Pressoir G. Brown P. J. Mitchell S. E. Rooney W. L. Tuinstra M. R. (2008). Community resources and strategies for association mapping in sorghum. Crop Sci. 48, 30–40. 10.2135/cropsci2007.02.0080

[B18] Cavanagh C. Morell M. Mackay I. Powell W. (2008). From mutations to MAGIC: resources for gene discovery, validation and delivery in crop plants. Curr. Opin. Plant Biol. 11 (2), 215–221. 10.1016/j.pbi.2008.01.002 18295532

[B19] Chen S. Zhou Y. Chen Y. Gu J. (2018). fastp: an ultra-fast all-in-one FASTQ preprocessor. Bioinformatics 34 (17), i884–i890. 10.1093/bioinformatics/bty560 30423086 PMC6129281

[B20] Dell’Acqua M. Gatti D. M. Pea G. Cattonaro F. Coppens F. Magris G. (2015). Genetic properties of the MAGIC maize population: a new platform for high-definition QTL mapping in Zea mays. Genome Biol. 16 (1), 167. 10.1186/s13059-015-0716-z 26357913 PMC4566846

[B21] DePristo M. A. Banks E. Poplin R. Garimella K. V. Maguire J. R. Hartl C. (2011). A framework for variation discovery and genotyping using next-generation DNA sequencing data. Nat. Genet. 43 (5), 491–498. 10.1038/ng.806 21478889 PMC3083463

[B22] Dickson S. P. Wang K. Krantz I. Hakonarson H. Goldstein D. B. (2010). Rare variants create synthetic genome-wide associations. PLoS Biol. 8 (1), e1000294. 10.1371/journal.pbio.1000294 20126254 PMC2811148

[B23] El Naim A. M. Mohammed K. E. Ibrahim E. A. Suleiman N. N. (2012). Impact of salinity on seed germination and early seedling growth of three sorghum (*Sorghum biolor* L. Moench) cultivars. Sci. Technol. 2 (2), 16–20. 10.5923/j.scit.20120202.03

[B24] Ewens W. J. Spielman R. S. (2001). Locating genes by linkage and association. Theor. Popul. Biol. 60 (3), 135–139. 10.1006/tpbi.2001.1547 11855947

[B25] Felderhoff T. J. Murray S. C. Klein P. E. Sharma A. Hamblin M. T. Kresovich S. (2012). QTLs for energy‐related traits in a sweet x grain sorghum [*Sorghum bicolor* (L.) Moench] mapping population. Crop Sci. 52 (5), 2040–2049. 10.2135/cropsci2011.11.0618

[B26] Feltus F. A. Hart G. E. Schertz K. F. Casa A. M. Brown P. Klein P. E. (2006). Alignment of genetic maps and QTLs between inter- and intra-specific sorghum populations. Theor. Appl. Genet. 112, 1295–1305. 10.1007/s00122-0060232-3 16491426

[B27] Flint‐Garcia S. A. Thuillet A. C. Yu J. Pressoir G. Romero S. M. Mitchell S. E. (2005). Maize association population: a high‐resolution platform for quantitative trait locus dissection. Plant J. 44 (6), 1054–1064. 10.1111/j.1365-313X.2005.02591.x 16359397

[B28] Girma G. Nida H. Seyoum A. Mekonen M. Nega A. Lule D. (2019). A large-scale genome-wide association analyses of Ethiopian sorghum landrace collection reveal loci associated with important traits. Front. Plant Sci. 10, 691. 10.3389/fpls.2019.00691 31191590 PMC6549537

[B29] Guindo D. Teme N. Vaksmann M. Doumbia M. Vilmus I. Guitton B. (2019). Quantitative trait loci for sorghum grain morphology and quality traits: toward breeding for a traditional food preparation of west-africa. J. Cereal Sci. 85, 256–272. 10.1016/j.jcs.2018.11.012

[B30] Harlan J. R. de Wet J. M. (1972). A simplified classification of cultivated sorghum 1. Crop Sci. 12 (2), 172–176. 10.2135/cropsci1972.0011183x001200020005x

[B31] Hart G. E. Schertz K. F. Peng Y. Syed N. H. (2001). Genetic mapping of *Sorghum bicolor* (L.) Moench QTLs that control variation in tillering and other morphological characters. Theor. Appl. Genet. 103, 1232–1242. 10.1007/s001220100582

[B32] Hashemi S. M. Perry G. Rajcan I. Eskandari M. (2022). SoyMAGIC: an unprecedented platform for genetic studies and breeding activities in soybean. Front. Plant Sci. 13, 945471. 10.3389/fpls.2022.945471 35874009 PMC9301248

[B33] Higgins R. H. Thurber C. S. Assaranurak I. Brown P. J. (2014). Multiparental mapping of plant height and flowering time QTL in partially isogenic sorghum families. G3 Genes, Genomes, Genet. 4 (9), 1593–1602. 10.1534/g3.114.013318 PMC4169151 25237111

[B34] Hilley J. L. Weers B. D. Truong S. K. McCormick R. F. Mattison A. J. McKinley B. A. (2017). Sorghum *Dw2* encodes a protein kinase regulator of stem internode length. Sci. Rep. 7 (1), 4616. 10.1038/s41598-017-04609-5 28676627 PMC5496852

[B35] Hmon K. P. W. Shehzad T. Okuno K. (2014). QTLs underlying inflorescence architecture in sorghum (*Sorghum bicolor* (L.) Moench) as detected by association analysis. Genet. Resour. Crop Evol. 61, 1545–1564. 10.1007/s10722-014-0129-y

[B36] Hmon K. P. W. Shehzad T. Okuno K. (2013). Variation in inflorescence architecture associated with yield components in a sorghum germplasm. Plant Genet. Resour. 11 (3), 258–265. 10.1017/s1479262113000154

[B37] Holland J. B. (2007). Genetic architecture of complex traits in plants. Curr. Opin. Plant Biol. 10 (2), 156–161. 10.1016/j.pbi.2007.01.003 17291822

[B38] Huang B. E. George A. W. Forrest K. L. Kilian A. Hayden M. J. Morell M. K. (2012). A multiparent advanced generation inter‐cross population for genetic analysis in wheat. Plant Biotechnol. J. 10 (7), 826–839. 10.1111/j.1467-7652.2012.00702.x 22594629

[B39] Huang B. E. Verbyla K. L. Verbyla A. P. Raghavan C. Singh V. K. Gaur P. (2015). MAGIC populations in crops: current status and future prospects. Theor. Appl. Genet. 128, 999–1017. 10.1007/s00122-015-2506-0 25855139

[B40] Huynh B. L. Ehlers J. D. Huang B. E. Muñoz‐Amatriaín M. Lonardi S. Santos J. R. (2018). A multi‐parent advanced generation inter‐cross (MAGIC) population for genetic analysis and improvement of cowpea (*Vigna unguiculata* L Walp). Plant J. 93 (6), 1129–1142. 10.1111/tpj.13827 29356213

[B41] Islam M. S. Thyssen G. N. Jenkins J. N. Zeng L. Delhom C. D. McCarty J. C. (2016). A MAGIC population-based genome-wide association study reveals functional association of GhRBB1_A07 gene with superior fiber quality in cotton. BMC Genomics 17 (1), 903–907. 10.1186/s12864-016-3249-2 27829353 PMC5103610

[B42] Jordan D. R. Mace E. S. Henzell R. G. Klein P. E. Klein R. R. (2010). Molecular mapping and candidate gene identification of the Rf2 gene for pollen fertility restoration in sorghum [*Sorghum bicolor* (L.) Moench]. Theor. Appl. Genet. 120, 1279–1287. 10.1007/s00122-009-1255-3 20091293

[B43] Kebede Y. (1991). “The role of Ethiopian sorghum germplasm resources in the national breeding programme,” in Plant genetic resources of Ethiopia (Cambridge: Cambridge University Press), 315–322.

[B44] Kilian A. Wenzl P. Huttner E. Carling J. Xia L. Blois H. (2012). Diversity arrays technology: a generic genome profiling technology on open platforms. Methods Mol. Biol. 888, 67–89. 10.1007/978-1-61779-870-2_5 22665276

[B45] Klein R. R. Mullet J. E. Jordan D. R. Miller F. R. Rooney W. L. Menz M. A. (2008). The effect of tropical sorghum conversion and inbred development on genome diversity as revealed by high‐resolution genotyping. Crop Sci. 48, S–12. 10.2135/cropsci2007.06.0319tpg

[B46] Kong W. Guo H. Goff V. H. Lee T. H. Kim C. Paterson A. H. (2014). Genetic analysis of vegetative branching in sorghum. Theor. Appl. Genet. 127, 2387–2403. 10.1007/s00122-014-2384-x 25163936

[B47] Korte A. Farlow A. (2013). The advantages and limitations of trait analysis with GWAS: a review. Plant Methods 9 (1), 29–9. 10.1186/1746-4811-9-29 23876160 PMC3750305

[B48] Kumar N. Boatwright J. L. Brenton Z. W. Sapkota S. Ballén-Taborda C. Myers M. T. (2023). Development and characterization of a sorghum multi-parent advanced generation intercross (MAGIC) population for capturing diversity among seed parent gene pool. G3 Genes, Genomes, Genet. 13 (4), jkad037. 10.1093/g3journal/jkad037 PMC10085790 36755443

[B49] Leiser W. L. Rattunde H. F. Weltzien E. Cisse N. Abdou M. Diallo A. (2014). Two in one sweep: aluminum tolerance and grain yield in P-limited soils are associated to the same genomic region in west african sorghum. BMC Plant Biol. 14, 206. 10.1186/s12870-014-0206-6 25112843 PMC4256928

[B50] Li H. Durbin R. (2009). Fast and accurate short read alignment with Burrows–Wheeler transform. Bioinformatics 25 (14), 1754–1760. 10.1093/bioinformatics/btp324 19451168 PMC2705234

[B51] Liu X. Huang M. Fan B. Buckler E. S. Zhang Z. (2016). Iterative usage of fixed and random effect models for powerful and efficient genome-wide association studies. PLoS Genet. 12 (2), e1005767. 10.1371/journal.pgen.1005767 26828793 PMC4734661

[B52] Mace E. S. Hunt C. H. Jordan D. R. (2013). Supermodels: sorghum and maize provide mutual insight into the genetics of flowering time. Theor. Appl. Genet. 126, 1377–1395. 10.1007/s00122-013-2059-z 23459955

[B53] Mace E. S. Innes D. Hunt C. Wang X. Tao Y. Baxter J. (2019). The sorghum QTL atlas: a powerful tool for trait dissection, comparative genomics and crop improvement. Theor. Appl. Genet. 132, 751–766. 10.1007/s00122-018-3212-5 30343386

[B54] Mackay I. Powell W. (2007). Methods for linkage disequilibrium mapping in crops. Trends Plant Sci. 12 (2), 57–63. 10.1016/j.tplants.2006.12.001 17224302

[B55] Mackay I. J. Bansept-Basler P. Barber T. Bentley A. R. Cockram J. Gosman N. (2014). An eight-parent multiparent advanced generation inter-cross population for winter-sown wheat: creation, properties, and validation. G3 Genes, Genomes, Genet. 4 (9), 1603–1610. 10.1534/g3.114.012963 PMC4169152 25237112

[B56] Mackay T. F. Stone E. A. Ayroles J. F. (2009). The genetics of quantitative traits: challenges and prospects. Nat. Rev. Genet. 10 (8), 565–577. 10.1038/nrg2612 19584810

[B57] MacQueen A. H. White J. W. Lee R. Osorno J. M. Schmutz J. Miklas P. N. (2020). Genetic associations in four decades of multi-environment trials reveal agronomic trait evolution in common bean. Genetics 215 (1), 267–284. 10.1534/genetics.120.303038 32205398 PMC7198278

[B58] Mangino G. Arrones A. Plazas M. Pook T. Prohens J. Gramazio P. (2022). Newly developed MAGIC population allows identification of strong associations and candidate genes for anthocyanin pigmentation in eggplant. Front. Plant Sci. 13, 847789. 10.3389/fpls.2022.847789 35330873 PMC8940277

[B59] McKenna A. Hanna M. Banks E. Sivachenko A. Cibulskis K. Kernytsky A. (2010). The genome analysis toolkit: a MapReduce framework for analyzing next-generation DNA sequencing data. Genome Res. 20 (9), 1297–1303. 10.1101/gr.107524.110 20644199 PMC2928508

[B60] Menz M. A. Klein R. R. Unruh N. C. Rooney W. L. Klein P. E. Mullet J. E. (2004). Genetic diversity of public inbreds of sorghum determined by mapped AFLP and SSR markers. Crop Sci. 44 (4), 1236–1244. 10.2135/cropsci2004.1236

[B61] Mindaye T. T. Mace E. S. Godwin I. D. Jordan D. R. (2015). Genetic differentiation analysis for the identification of complementary parental pools for sorghum hybrid breeding in Ethiopia. Theor. Appl. Genet. 128 (9), 1765–1775. 10.1007/s00122-015-2545-6 26024715

[B62] Morris G. P. Ramu P. Deshpande S. P. Hash C. T. Shah T. Upadhyaya H. D. (2013). Population genomic and genome-wide association studies of agroclimatic traits in sorghum. Proc. Natl. Acad. Sci. 110 (2), 453–458. 10.1073/pnas.1215985110 23267105 PMC3545811

[B63] Murray S. C. Rooney W. L. Hamblin M. T. Mitchell S. E. Kresovich S. (2009). Sweet sorghum genetic diversity and association mapping for brix and height. Plant Genome 2, 11. 10.3835/plantgenome2008.10.0011

[B64] Olatoye M. O. Hu Z. Morris G. P. (2020). Genome‐wide mapping and prediction of plant architecture in a sorghum nested association mapping population. Plant Genome 13 (3), e20038. 10.1002/tpg2.20038 33217207 PMC12806869

[B65] Ongom P. O. Ejeta G. (2018). Mating design and genetic structure of a multi-parent advanced generation intercross (MAGIC) population of sorghum (*Sorghum bicolor* L. Moench). G3 Genes, Genomes, Genet. 8, 331–341. 10.1534/g3.117.300248 PMC5765360 29150594

[B66] Parra-Londono S. Fiedler K. Kavka M. Samans B. Wieckhorst S. Zacharias A. (2018). Genetic dissection of early-season cold tolerance in sorghum: genome-wide association studies for seedling emergence and survival under field and controlled environment conditions. Theor. Appl. Genet. 131, 581–595. 10.1007/s00122-017-3021-2 29147737

[B67] Pascual L. Desplat N. Huang B. E. Desgroux A. Bruguier L. Bouchet J. P. (2015). Potential of a tomato MAGIC population to decipher the genetic control of quantitative traits and detect causal variants in the resequencing era. Plant Biotechnol. J. 13 (4), 565–577. 10.1111/pbi.12282 25382275

[B68] Patil N. Y. Pugh N. A. Klein R. R. Martinez H. S. Martinez R. S. Rodriguez-Herrera R. (2019). Heritability and quantitative trait loci of composition and structural characteristics in sorghum grain. J. Crop Improv. 33 (1), 1–24. 10.1080/15427528.2018.1536006

[B69] Poplin R. Ruano-Rubio V. DePristo M. A. Fennell T. J. Carneiro M. O. Van der Auwera G. A. (2018). Scaling accurate genetic variant discovery to tens of thousands of samples. BioRxiv 14 201178.

[B70] R Foundation (2020). R: A language and environment for statistical computing. Vienna, Austria: R Foundation for Statistical Computing. Available at: https://www.R-project.org/ .

[B71] Reddy N. R. Madhusudhana R. Murali Mohan S. Chakravarthi D. V. Mehtre S. P. Seetharama N. (2013). Mapping QTL for grain yield and other agronomic traits in post-rainy sorghum [*Sorghum bicolor* (L.) Moench]. Theor. Appl. Genet. 126 (8), 1921–1939. 10.1007/s00122-013-2107-8 23649648

[B72] Reddy N. R. Ragimasalawada M. Sabbavarapu M. M. Nadoor S. Patil J. V. (2014). Detection and validation of stay-green QTL in post-rainy sorghum involving widely adapted cultivar, M35-1 and a popular stay-green genotype B35. BMC Genomics 15, 1–6. 10.1186/1471-2164-15-909 25326366 PMC4219115

[B73] Rooney W. L. (2004). Sorghum improvement-integrating traditional and new technology to produce improved genotypes. Adv. Agron. 83, 37–109. 10.1016/s0065-2113(04)83002-5

[B74] Sabadin P. K. Malosetti M. Boer M. P. Tardin F. D. Santos F. G. Guimaraes C. T. (2012). Studying the genetic basis of drought tolerance in sorghum by managed stress trials and adjustments for phenological and plant height differences. Theor. Appl. Genet. 124, 1389–1402. 10.1007/s00122-012-1795-9 22297563

[B75] Sallam A. Martsch R. (2015). Association mapping for frost tolerance using multi-parent advanced generation inter-cross (MAGIC) population in faba bean (*Vicia faba* L). Genetica 143 (4), 501–514. 10.1007/s10709-015-9848-z 26041397

[B76] Sangma B. (2013). “Genetic characterization of flowering time in sorghum,”. Ph.D. Thesis, School of Agriculture and Food Sciences (Australia: The University of Queensland).

[B77] Sannemann W. Huang B. E. Mathew B. Léon J. (2015). Multi-parent advanced generation inter-cross in barley: high-resolution quantitative trait locus mapping for flowering time as a proof of concept. Mol. Breed. 35, 86–6. 10.1007/s11032-015-0284-7

[B78] Schertz K. F. Sivaramakrishnan S. Hanna W. W. Mullet J. Sun Y. Murty U. R. (1997). “Alternate cytoplasms and apomixis of sorghum and pearl millet,” in Proceedings of the international conference on genet. Improvement of sorghum and pearl millet (Lubbock, Texas, USA: Texas A&M University), 213–223.

[B79] Scott M. F. Ladejobi O. Amer S. Bentley A. R. Biernaskie J. Boden S. A. (2020). Multi-parent populations in crops: a toolbox integrating genomics and genetic mapping with breeding. Heredity 125, 396–416. 10.1038/s41437-020-0336-6 32616877 PMC7784848

[B80] Shehzad T. Okuno K. (2015). QTL mapping for yield and yield-contributing traits in sorghum (*Sorghum bicolor* (L.) Moench) with genome-based SSR markers. Euphytica 203, 17–31. 10.1007/s10681-014-1243-9

[B81] Stadlmeier M. Hartl L. Mohler V. (2018). Usefulness of a multiparent advanced generation intercross population with a greatly reduced mating design for genetic studies in winter wheat. Fronters Plant Sci. 871, 1–12. 10.3389/fpls.2018.01825 PMC6291512 30574161

[B82] Stephens M. (2017). False discovery rates: a new deal. Biostatistics 18 (2), 275–294. 10.1093/biostatistics/kxw041 27756721 PMC5379932

[B83] Takai T. Yonemaru J. I. Kaidai H. Kasuga S. (2012). Quantitative trait locus analysis for days-to-heading and morphological traits in an RIL population derived from an extremely late flowering F_1_ hybrid of sorghum. Euphytica 187, 411–420. 10.1007/s10681-012-0727-8

[B84] Unterseer S. Bauer E. Haberer G. Seidel M. Knaak C. Ouzunova M. (2014). A powerful tool for genome analysis in maize: development and evaluation of the high density 600 k SNP genotyping array. BMC Genomics 15 (1), 823–825. 10.1186/1471-2164-15-823 25266061 PMC4192734

[B85] Urbut S. M. Wang G. Carbonetto P. Stephens M. (2019). Flexible statistical methods for estimating and testing effects in genomic studies with multiple conditions. Nat. Genet. 51 (1), 187–195. 10.1038/s41588-018-0268-8 30478440 PMC6309609

[B86] Van der Auwera G. A. Carneiro M. O. Hartl C. Poplin R. Del Angel G. Levy‐Moonshine A. (2013). From FastQ data to high‐confidence variant calls: the genome analysis toolkit best practices pipeline. Curr. Protoc. Bioinforma. 43 (1), 11. 10.1002/0471250953.bi1110s43 PMC4243306 25431634

[B87] Vilhjálmsson B. J. Nordborg M. (2013). The nature of confounding in genome-wide association studies. Nat. Rev. Genet. 14 (1), 1–2. 10.1038/nrg3382 23165185

[B88] Wang L. Upadhyaya H. D. Zheng J. Liu Y. Singh S. K. Gowda C. L. (2021). Genome-Wide association mapping identifies novel panicle morphology loci and candidate genes in sorghum. Front. Plant Sci. 12, 743838. 10.3389/fpls.2021.743838 34675951 PMC8525895

[B89] Wang Q. Tian F. Pan Y. Buckler E. S. Zhang Z. (2014a). A SUPER powerful method for genome wide association study. PLoS One 9 (9), e107684. 10.1371/journal.pone.0107684 25247812 PMC4172578

[B90] Wang X. Mace E. Hunt C. Cruickshank A. Henzell R. Parkes H. (2014b). Two distinct classes of QTL determine rust resistance in sorghum. BMC Plant Biol. 14, 366. 10.1186/s12870-014-0366-4 25551674 PMC4335369

[B91] Xin Z. Wang M. Cuevas H. E. Chen J. Harrison M. Pugh N. A. (2021). Sorghum genetic, genomic, and breeding resources. Planta 254 (6), 114. 10.1007/s00425-021-03742-w 34739592 PMC8571242

[B92] Yin L. Zhang H. Tang Z. Xu J. Yin D. Zhang Z. (2021). rMVP: A memory-efficient, visualization-enhanced, and parallel-accelerated tool for genome-wide association study. Proteomics Bioinforma. 19 (4), 619–628. 10.1016/j.gpb.2020.10.007 PMC9040015 33662620

[B93] Zhang C. Dong S. S. Xu J. Y. He W. M. Yang T. L. (2019). PopLDdecay: a fast and effective tool for linkage disequilibrium decay analysis based on variant call format files. Bioinformatics 35 (10), 1786–1788. 10.1093/bioinformatics/bty875 30321304

[B94] Zhang D. Kong W. Robertson J. Goff V. H. Epps E. Kerr A. (2015). Genetic analysis of inflorescence and plant height components in sorghum (Panicoidae) and comparative genetics with rice (Oryzoidae). BMC Plant Biol. 15 (1), 107. 10.1186/s12870-015-0477-6 25896918 PMC4404672

[B95] Zhang Z. Ersoz E. Lai C. Q. Todhunter R. J. Tiwari H. K. Gore M. A. (2010). Mixed linear model approach adapted for genome-wide association studies. Nat. Genet. 42 (4), 355–360. 10.1038/ng.546 20208535 PMC2931336

[B96] Zhou Y. Srinivasan S. Mirnezami S. V. Kusmec A. Fu Q. Attigala L. (2019). Semiautomated feature extraction from RGB images for sorghum panicle architecture GWAS. Plant Physiol. 179 (1), 24–37. 10.1104/pp.18.00974 30389784 PMC6324233

