# Supplementary material for: Discovering useful genetic variation in the seed parent gene pool for sorghum improvement
Source: Front Genet. 2023 Sep 18;14:1221148. doi: 10.3389/fgene.2023.1221148 (PMC10544336; doi:10.3389/fgene.2023.1221148)
Supplement: Supplementary file 1 [file DataSheet2.pdf]

# *Supplementary Material*

## Discovering Useful Genetic Variation in the Seed Parent Gene Pool for Sorghum Improvement

Neeraj Kumar[1,2,†,*], J. Lucas Boatwright[1,2,†], Sirjan Sapkota[1], Zachary W. Brenton[1,3], Carolina Ballén-Taborda[2,4], Matthew T. Myers[1,2], William A. Cox[1,2], Kathleen E. Jordan[1,2], Stephen Kresovich[1,2,5], Richard E. Boyles[2,4,*]

[1]Advanced Plant Technology, Clemson University, Clemson, SC 29634, [2]Department of Plant and Environmental Sciences, Clemson University, Clemson, SC 29634, [3]Carolina Seed Systems, Darlington, SC 29532, [4]Pee Dee Research and Education Center, Clemson University, Florence, SC 29506, [5]Feed the Future Innovation Lab for Crop Improvement, Cornell University, Ithaca, NY, USA, [†]These authors contributed equally to this work. [*]Corresponding authors.

Scripts for data analysis are available on GitHub (https://github.com/jlboat/SorghumMagic) under MIT license.
