# Supplementary material for: Discovering useful genetic variation in the seed parent gene pool for sorghum improvement
Source: Front Genet. 2023 Sep 18;14:1221148. doi: 10.3389/fgene.2023.1221148 (PMC10544336; doi:10.3389/fgene.2023.1221148)
Supplement: Supplementary file 2 [file DataSheet1.pdf]

Supplementary Material

Discovering Useful Genetic Variation in the Seed Parent Gene Pool for Sorghum Improvement

Neeraj Kumar[1,2,†,*], J. Lucas Boatwright[1,2,†], Sirjan Sapkota[1], Zachary W. Brenton[1,3], Carolina Ballén-Taborda[2,4], Matthew T. Myers[1,2], William A. Cox[1,2], Kathleen E. Jordan[1,2], Stephen Kresovich[1,2,5], Richard E. Boyles[2,4,*]

[1]Advanced Plant Technology, Clemson University, Clemson, SC 29634, [2]Department of Plant and Environmental Sciences, Clemson University, Clemson, SC 29634, [3]Carolina Seed Systems, Darlington, SC 29532, [4]Pee Dee Research and Education Center, Clemson University, Florence, SC 29506, [5]Feed the Future Innovation Lab for Crop Improvement, Cornell University, Ithaca, NY, USA, [†]These authors contributed equally to this work. [*]Corresponding authors.

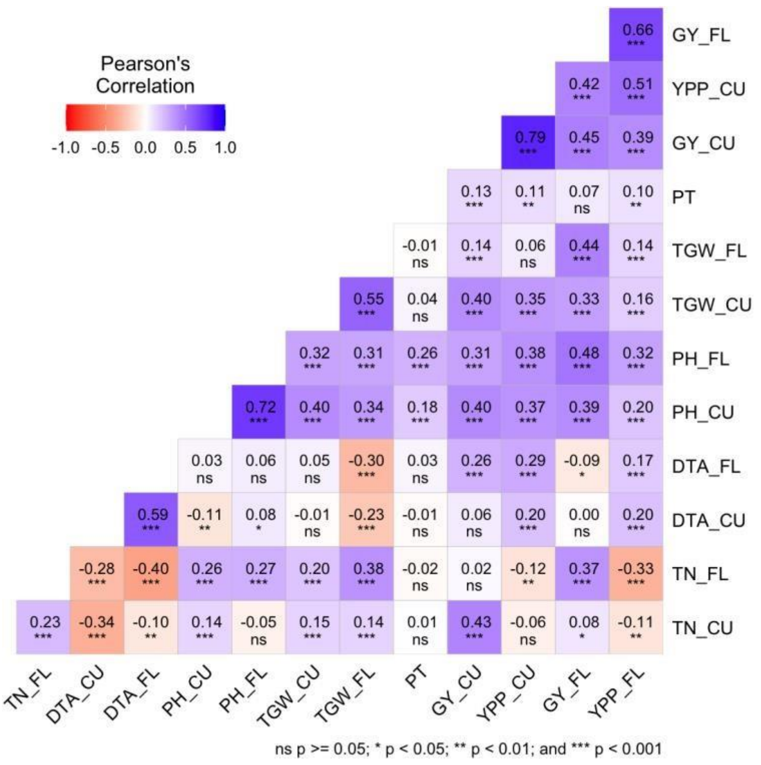

Supplementary Figure S1 Pearson's correlation coefficients between two locations (FL = Florence and CU = Clemson University) of each phenotypic traits; PT: panicle type, DTA: days to anthesis, PH: plant height, GY: grain yield, TGW: 1000-grain weight, TN: tiller number per meter and YPP: yield per panicle. Panicle type (PT) was scored only at CU location.

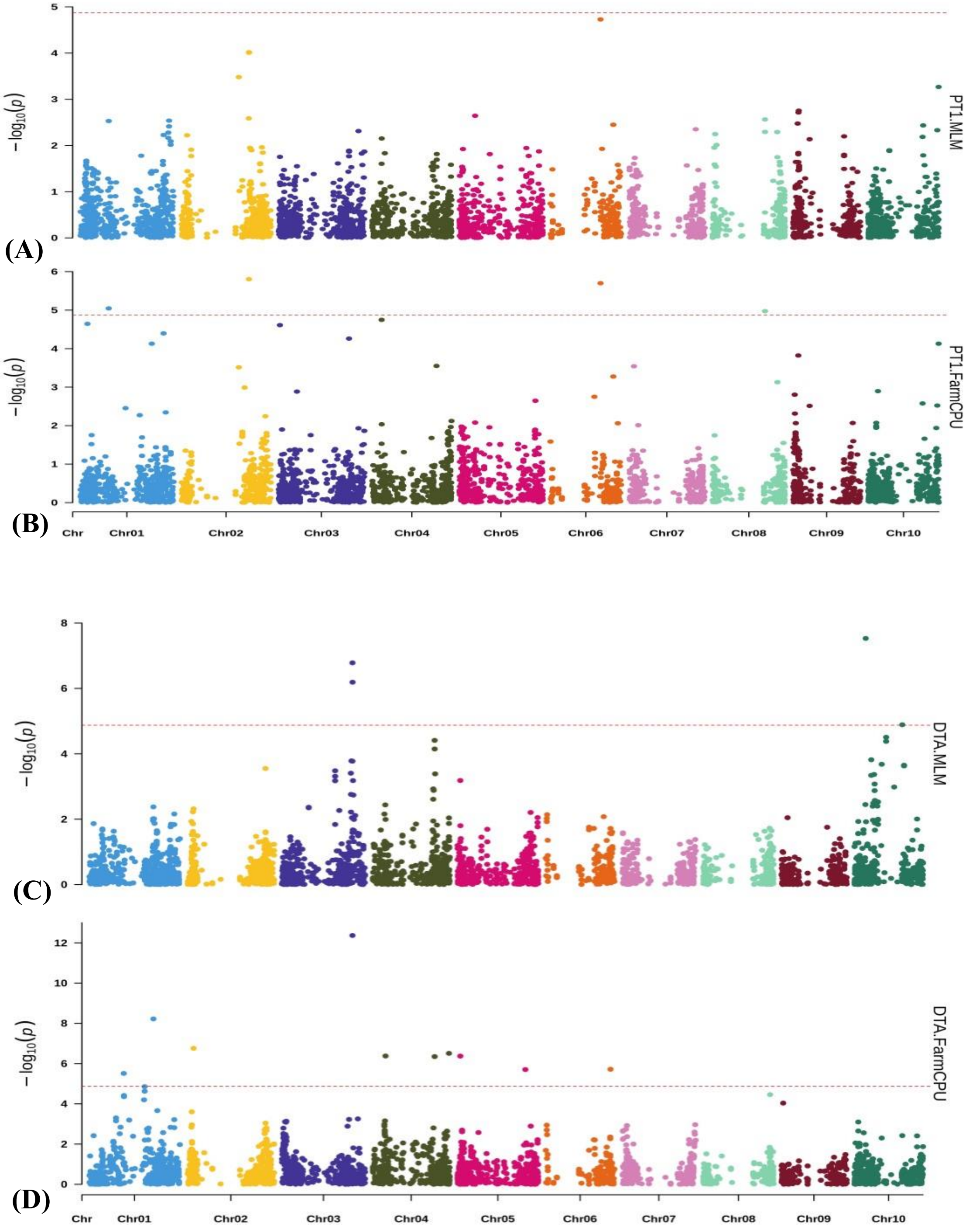
(A)
PT1.MLM
(B)
PT1.FarmCPU
Chr Chr01 Chr02 Chr03 Chr04 Chr05 Chr06 Chr07 Chr08 Chr09 Chr10
(C)
DTA.MLM
(D)
DTA.FarmCPU
Chr Chr01 Chr02 Chr03 Chr04 Chr05 Chr06 Chr07 Chr08 Chr09 Chr10
$-\log_{10}(p)$

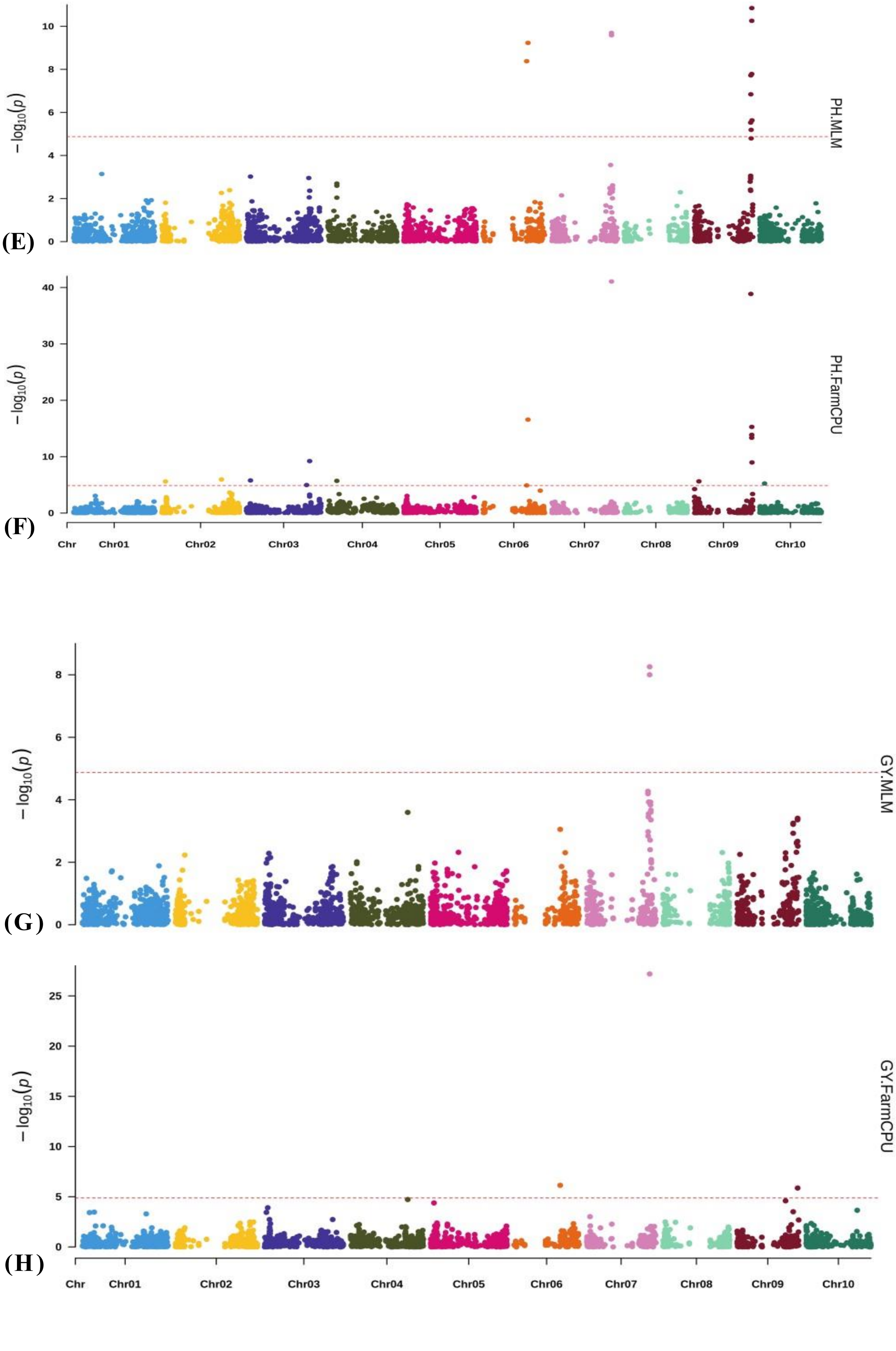

(E)
PH.MLM
(F)
PH.FarmCPU
(G)
GY.MLM
(H)
GY.FarmCPU
$-\log_{10}(p)$
Chr
Chr01
Chr02
Chr03
Chr04
Chr05
Chr06
Chr07
Chr08
Chr09
Chr10

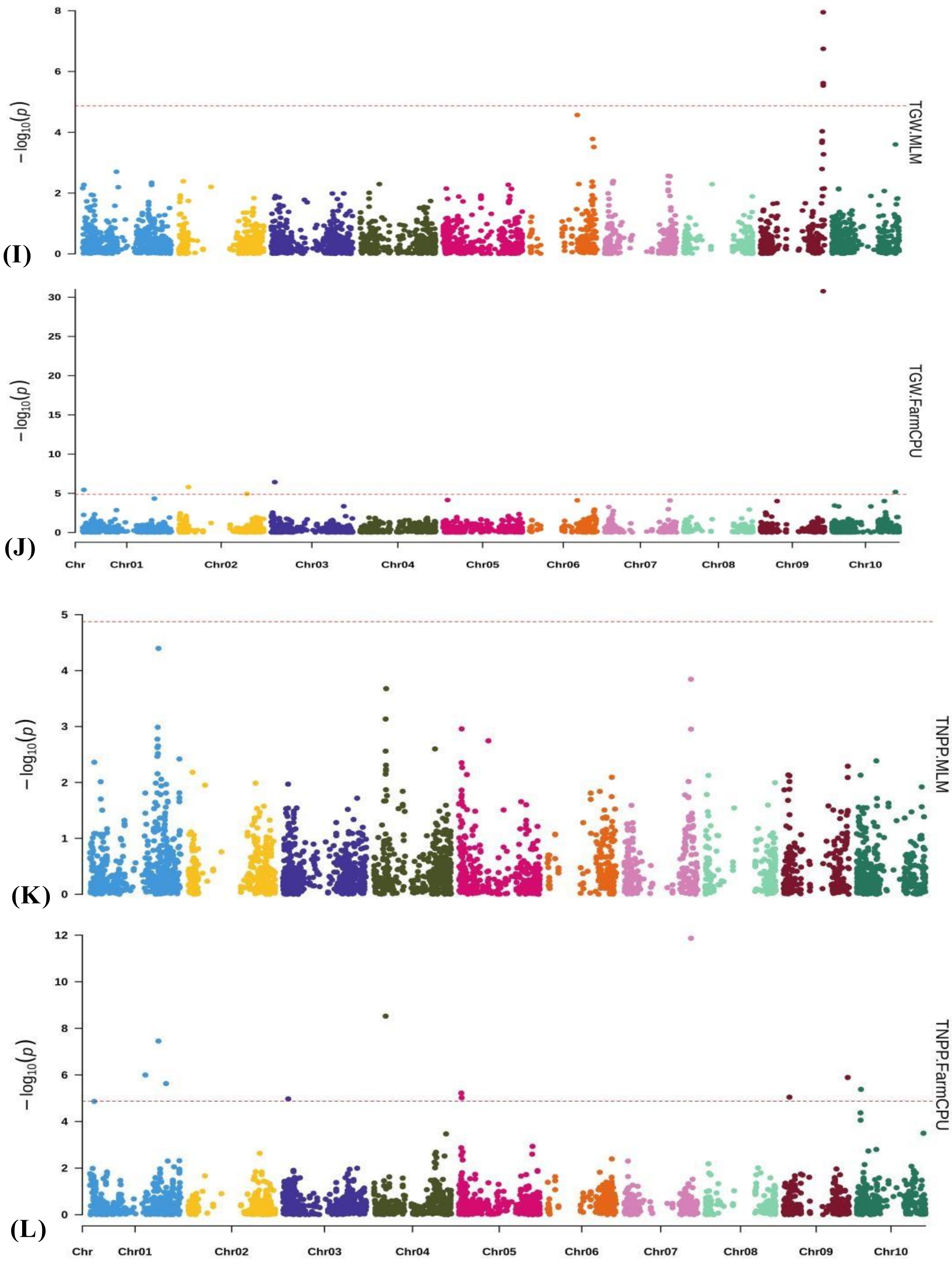

TGW.MLM
TGW.FarmCPU
TNPP.MLM
TNPP.FarmCPU
$-\log_{10}(p)$
(I)
(J)
(K)
(L)
Chr
Chr01
Chr02
Chr03
Chr04
Chr05
Chr06
Chr07
Chr08
Chr09
Chr10

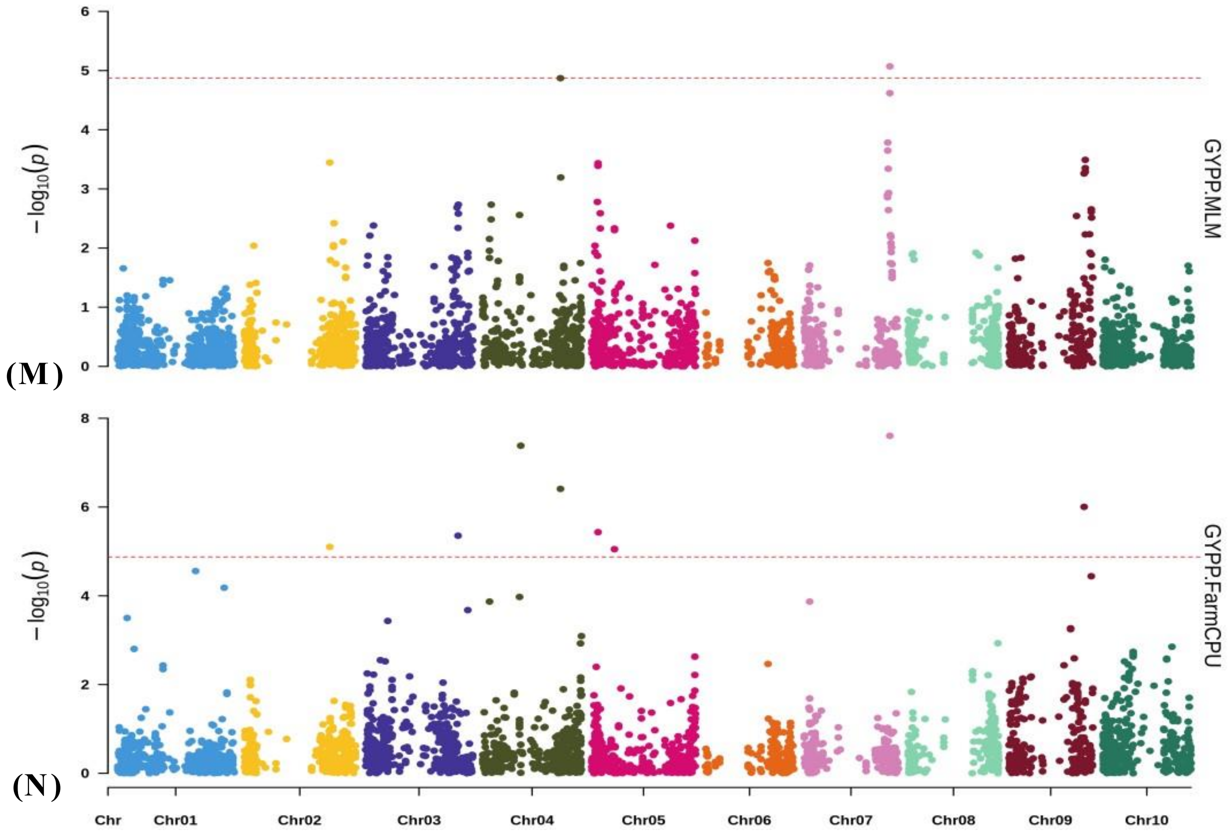

Supplementary Figure S2 Manhattan plots based on *rMVP* GWAS program following two models (MLM and FarmCPU) using MBL population. Plots A, C, E, G, I, K, and M are based on MLM model, whereas plots B, D, F, H, J, L, and N are based on FarmCPU model. (A and B) panicle type (PT), (C and D) days to anthesis (DTA), (E and F) plant height (PH), (G and H) grain yield (GY), (I and J) 1000-grain weight (TGW), (K and L) tiller number per meter (TN), and (M and N) yield per panicle (YPP). The $-\log_{10}(p)$ values (*y*-axis) are plotted against the position on each chromosome (*x*-axis). Each solid circle represents a SNP, and the red dashed line represents the Bonferroni-corrected threshold ($p \leq 0.05$).

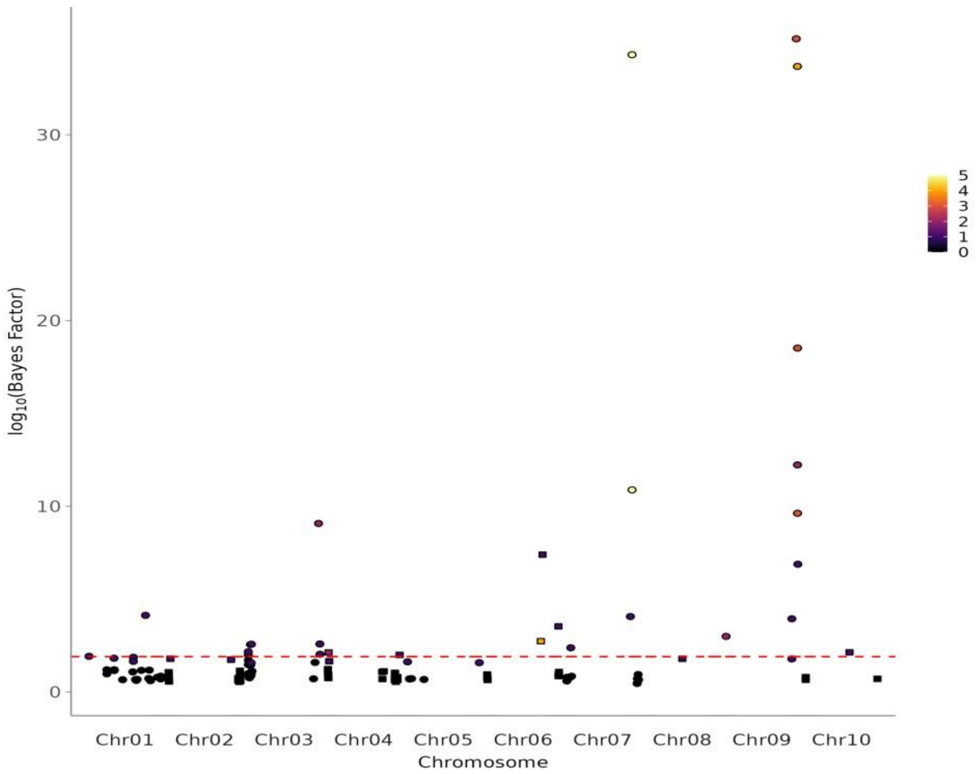

(A) Significant SNPs with pleiotropic effects identified using FarmCPU model.

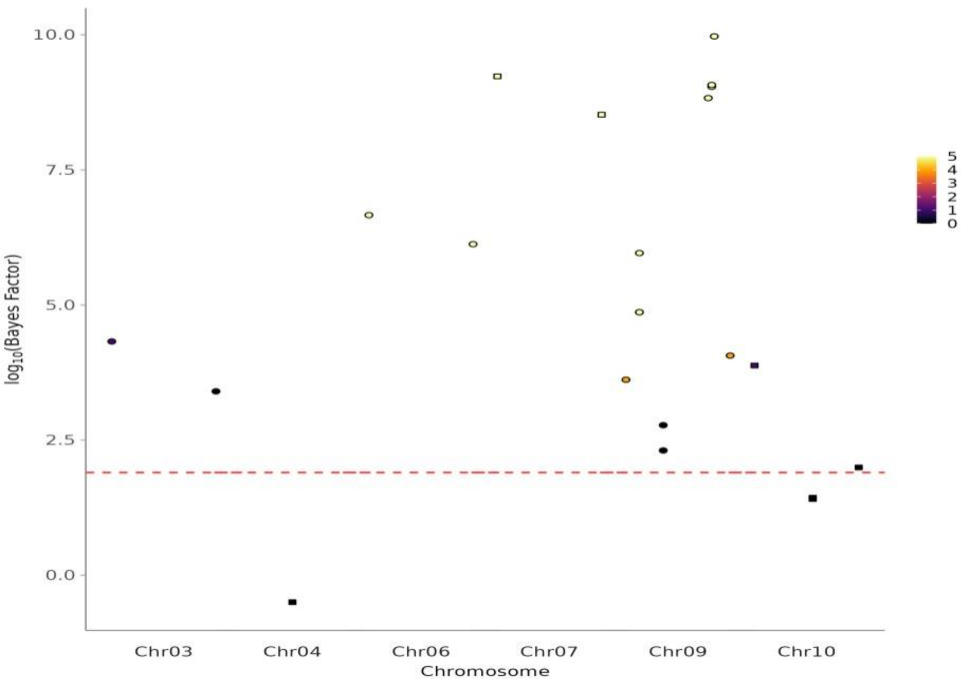

(B) Significant SNPs with pleiotropic effects identified using MLM model.

Supplementary Figure S3: Plot of the significant SNPs identified using *mashr* program for various traits in MBL population.

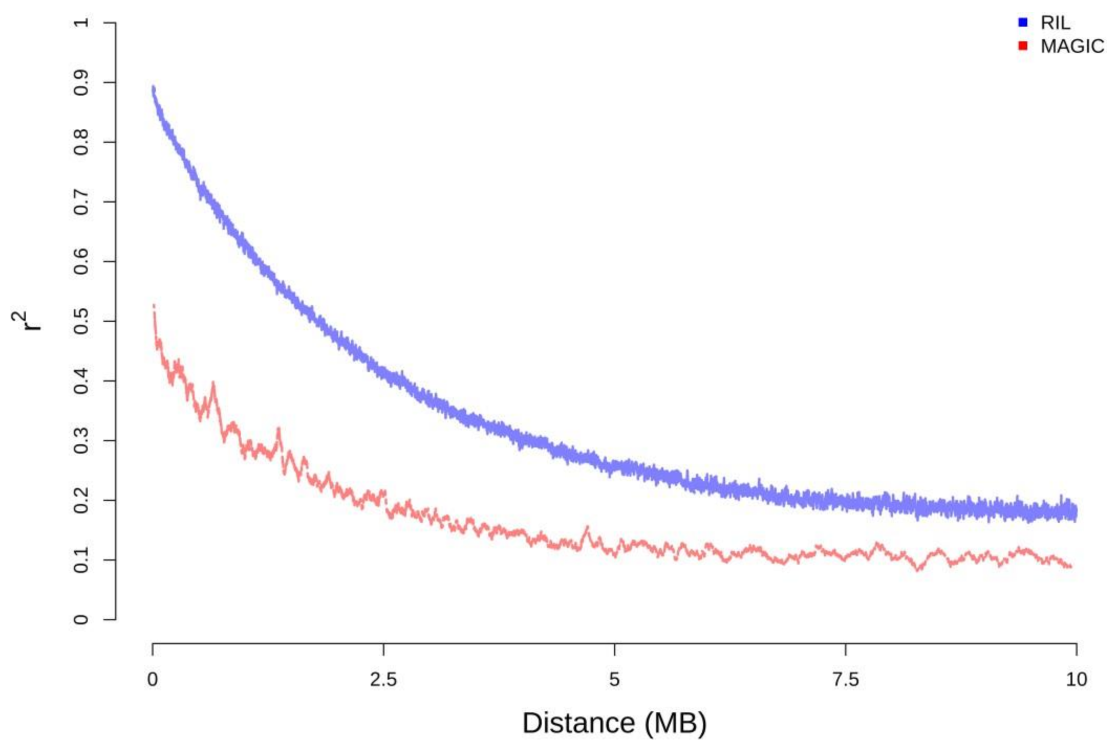

Supplementary Figure S4. Linkage decay (Pearson's correlation coefficient squared) of MBL and a recombinant inbred line (RIL) populations plotted against the distance in Mega base (Mb) across the genome. RIL mapping population sharing two parents of MBL (BTxARG-1 and BTx642) was earlier published by Boyles et al. (2017b).
